# Plasmonic imaging of the layer-dependent electrocatalytic activity of two-dimensional catalysts

**DOI:** 10.1038/s41467-022-35633-3

**Published:** 2022-12-22

**Authors:** Xiaona Zhao, Xiao-Li Zhou, Si-Yu Yang, Yuan Min, Jie-Jie Chen, Xian-Wei Liu

**Affiliations:** 1 Chinese Academy of Sciences Key Laboratory of Urban Pollutant Conversion, Department of Environmental Science and Engineering, University of Science and Technology of China, Hefei, 230026 China; 2 School of Environmental and Biological Engineering, Nanjing University of Science and Technology, Nanjing, 210094 China; 3 Department of Applied Chemistry, University of Science and Technology of China, Hefei, 230026 China

**Keywords:** Heterogeneous catalysis, Electrocatalysis, Imaging studies, Imaging and sensing

## Abstract

Studying the localized electrocatalytic activity of heterogeneous electrocatalysts is crucial for understanding electrocatalytic reactions and further improving their performance. However, correlating the electrocatalytic activity with the microscopic structure of two-dimensional (2D) electrocatalysts remains a great challenge due to the lack of in situ imaging techniques and methods of tuning structures with atomic precision. Here, we present a general method of probing the layer-dependent electrocatalytic activity of 2D materials in situ using a plasmonic imaging technique. Unlike the existing methods, this approach was used to visualize the surface charge density and electrocatalytic activity of single 2D MoS_2_ nanosheets, enabling the correlation of layer-dependent electrocatalytic activity with the surface charge density of single MoS_2_ nanosheets. This work provides insights into the electrocatalytic mechanisms of 2D transition metal dichalcogenides, and our approach can serve as a promising platform for investigating electrocatalytic reactions at the heterogeneous interface, thus guiding the rational design of high-performance electrocatalysts.

## Abstract (web summary)

Probing the localized electrocatalytic activity of heterogeneous electrocatalysts is crucial. Here, the authors propose a method of imaging the surface charge density and electrocatalytic activity of single two-dimensional electrocatalyst nanosheets.

## Introduction

Heterogeneous electrocatalysis plays pivotal roles in various renewable energy conversion technologies^1–3^. Quantitative characterization of the localized electrocatalytic activity of heterogeneous electrocatalysts is crucial for understanding electrochemical reactions and further improving their performance. For example, two-dimensional (2D) nanomaterials are promising candidates for electrocatalytic hydrogen evolution reactions due to their high electrochemical performance^4–6^. Correlating the electrocatalytic activity of two-dimensional electrocatalysts with their microscopic structure can guide the design of ideal catalyst materials.

Although substantial work has been accomplished in characterizing the macroscopic catalytic activity of 2D electrocatalysts, these ensemble measurements based on numerous nanosheets limit our understanding of the spatial heterogeneity of catalysis^7–9^. Several techniques have been used to map the electrocatalytic activity of single nanosheets of 2D materials at the nanosheet-liquid interface, including scanning electrochemical microscopy (SECM)^10–13^, scanning ion conductance microscopy (SICM)^14,15^, and single molecule fluorescence microscopy^16–18^. These approaches offer high spatial resolution. However, the imaging speed is limited, hindering the acquisition of real-time and in situ information during electrochemical reactions. More importantly, a complete electrochemical process includes charge transfer from the electrode to the surface of the electrocatalyst. The electron transport among layers in a single nanosheet also plays a vital role in determining the electrocatalytic performance^19,20^. However, imaging the electrocatalytic activity of 2D electrocatalysts in situ and therefore correlating the electrocatalytic activity with their layer structures remains experimentally challenging.

**Fig. 1:**
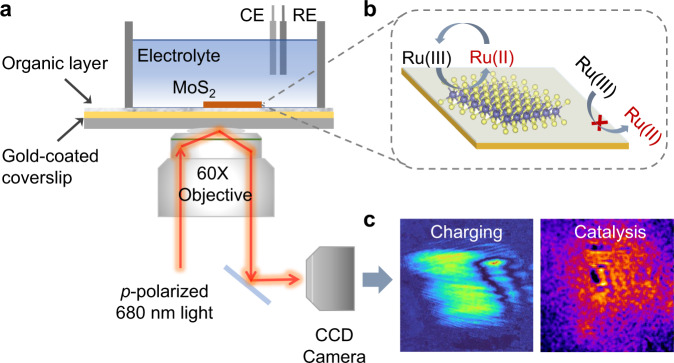
Plasmonic imaging of the electrochemical reactions of single MoS_2_ nanosheets. **a** Experimental setup. **b** Schematic depicting the removal of the interference from the gold film, which was passivated by a self-assembled monolayer (SAM) of 1-octadecanethiol molecules. Ru(III) and Ru(II) represent [Ru(NH_3_)_6_]^3+^ and [Ru(NH_3_)_6_]^2+^, respectively. **c** Typical images displaying the surface charge density and electrocatalytic activity of monolayer MoS_2_.

Here, we report plasmonic imaging of the layer-dependent electrocatalytic activity of 2D transition metal dichalcogenide (TMD) nanosheets, including MoS_2_, WS_2_, MoSe_2_, and graphene (Fig. 1a). The plasmonic-based electrochemical current imaging approach measures the electrochemical reactions of nanoparticles on a gold film with a high temporal resolution^21,22^. Although there has been intensive interests in mapping the electrocatalytic activity of 2D nanomaterials, the large background signal arising from the gold film interferes with the electrochemical reaction signals^23–25^, which limits the application of plasmonic imaging in 2D nanomaterials. We therefore propose a strategy to eliminate the interference (Fig. 1b) and distinguish and quantify the surface charge density and electrocatalytic activity of single TMD nanosheets (Fig. 1c); in this strategy, MoS_2_ was used as an example. Furthermore, by integrating our developed method of in situ fabrication of the desired layer number of TMDs with surface plasmon etching^26^, we precisely visualized the layer-dependent electrocatalytic activity of the same MoS_2_ nanosheet in real time, which is a difficult task with other techniques. Thus, the obtained knowledge can provide additional insights into the origin of the underlying mechanisms during electrocatalysis, and this optical imaging technique can be used in measuring the charge transport, defect distribution and electrocatalytic activity of other TMDs.

## Results

### Mapping the surface charge density of monolayer MoS_2_

To excite surface plasmons, we used a home-built inverted microscope (Nikon Ti-E; Japan) with a high numerical aperture (NA = 1.49) oil-immersion objective (Fig. 1a). A beam of *p*-polarized incident light was collimated to illuminate a gold film with a certain incident angle. The reflected light was collected by a high-speed camera to acquire plasmonic images. The MoS_2_ monolayers were fabricated in situ by a surface plasmon-driven etching method recently developed by our group^26^. We therefore employed this bifunctional setup to fabricate MoS_2_ monolayers and image the electrocatalytic activity of monolayer MoS_2_. To block the background charging signal of the gold film, we passivated the gold-coated coverslip with a self-assembled monolayer (SAM) of 1-octadecanethiol molecules. After the mechanically exfoliated MoS_2_ multilayers were transferred onto the SAM-coated gold film, we directly converted them into corresponding monolayers while keeping their original lateral size, which was validated by Raman spectroscopy, with the separation between the A_1g_ and E_2g_ peaks decreasing from 25.1 to 20.3 cm^−1^ (Supplementary Fig. 1). We employed a monolayer MoS_2_-deposited gold film as the working electrode, a platinum wire as the counter electrode and Ag/AgCl as the reference electrode in the three-electrode electrochemical cell.

**Fig. 2:**
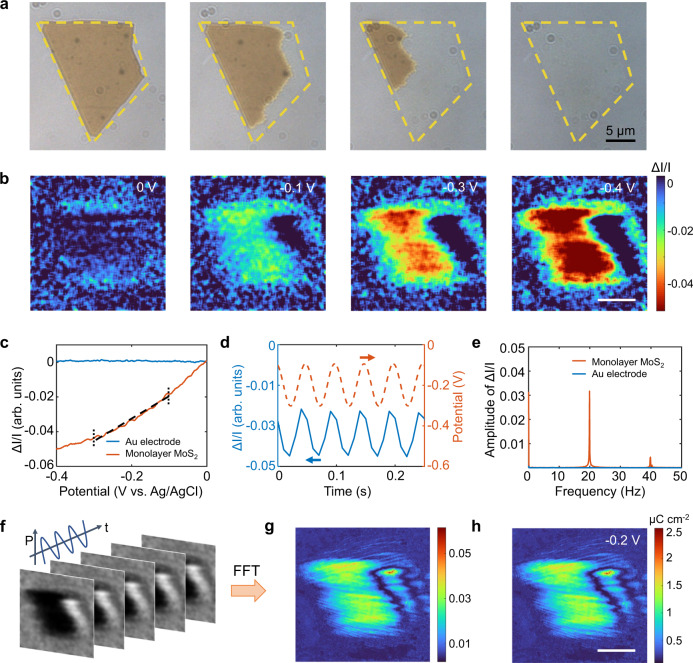
Potential modulation of the surface charge density of monolayer MoS_2_. **a** Optical image of monolayer MoS_2_ on a 1-octadecanethiol-modified gold film. **b** Plasmonic images of monolayer MoS_2_ at potentials of 0 V, −0.1 V, −0.3 V and −0.4 V in 260 mM phosphate buffer (pH = 6.86). Each of the images was obtained by subtracting the first image at 0 V. Scale bar: 10 μm. **c** Potential dependence of the plasmonic intensity change ($\triangle I/I$) of monolayer MoS_2_ and the gold film. **d** Applied potential modulation (dashed line) and the resultant plasmonic intensity change of monolayer MoS_2_. **e** FFT spectra of monolayer MoS_2_ (blue line) and the Au electrode (red line). **f** Time sequence of plasmonic images of monolayer MoS_2_ recorded at 100 frames/s with a sinusoidal potential modulation amplitude of 100 mV and frequency of 20 Hz. **g** FFT image determined by performing FFT on each pixel of the optical images in the time domain. **h** Surface charge density image of monolayer MoS_2_ at a potential of −0.2 V, which was acquired by converting the FFT image in (**g**) with a calibration factor.

As the plasmonic imaging technique is sensitive to surface charge density^23,27^, we examined the plasmonic intensity dependence of monolayer MoS_2_ on the surface charge variations before measuring the electrochemical activity. The potential of monolayer MoS_2_ on a modified gold film was modulated in the range of 0 V and −0.4 V (vs. Ag/AgCl) in 260 mM phosphate buffer solution (PB). Figure 2a shows an optical image of monolayer MoS_2_. We obtained difference plasmonic images of monolayer MoS_2_ by subtracting the plasmonic image captured at 0 V from each of the subsequent frames during cycling the potential scanning. Figure 2b exhibits several snapshots of the plasmonic images of monolayer MoS_2_ at different potentials, showing a significant increase in the image contrast during the cathodic scan of cyclic voltammetry (CV). The coating of the gold film with 1-octadecanethiol molecules can block background current from the gold substrate, while electrons can tunnel from the gold film to the monolayer MoS_2_ through the molecular layer with negligible resistance^28,29^. The electron density of monolayer MoS_2_ increased with decreasing potential, causing a shift in its dielectric constant, which was responsible for the observed increase in the plasmonic image contrast (see Supplementary Note 2 and Supplementary Fig. 2 for details). Consequently, we observed that the plasmonic intensity change ($\triangle I/I$) of monolayer MoS_2_ decreased rapidly as the potential swept negatively from 0 V to −0.4 V (Fig. 2c). However, the plasmonic intensity of the Au electrode remained constant over the same potential window.

To quantify the charge dependence of the plasmonic intensity, we performed sinusoidal potential modulation on monolayer MoS_2_ and captured time-dependent images (see Supplementary Note 3 and Supplementary Fig. 3 for details). The plasmonic intensity versus time presents periodic intensity oscillation (blue line, Fig. 2d) with a synchronous change relative to the applied potential (red dash, Fig. 2d). We conducted a fast Fourier transform (FFT) on the time-domain plasmonic intensity and observed an obvious peak at 20 Hz for monolayer MoS_2_, consistent with the frequency of the applied potential (red line, Fig. 2e). There was no response for the gold film (blue line, Fig. 2e), indicating that the observed plasmonic intensity response was derived from monolayer MoS_2_. By performing FFT on the plasmonic intensity of each pixel in time sequence images, we extracted the amplitude at the frequency of the modulation potential for each pixel and constructed an FFT image (Fig. 2f, g). The relationship between the surface charge density of monolayer MoS_2_ ($\triangle q$) and the plasmonic intensity $\triangle I/I$ is

1 $$\triangle q=A\,\triangle I/I$$

where *A* is a calibration factor, which can be calculated from the slope of the fitting curve (Fig. 2c). To obtain an accurate calibration factor, the slope $(\triangle I/I)/\Delta V$ was determined to be 0.12 V^–1^ from the same potential window (−0.3 V to −0.1 V); $\triangle V$ is the modulation potential. Since $\Delta q=c\Delta V$, the calibration factor can be expressed as: $A=c\Delta V/(\triangle I/I)$, where *c* is the capacitance per unit area, which is determined to be ∼5 μF cm^−2^ for monolayer MoS_2_ (see Supplementary Note 4 and Supplementary Fig. 4 for details)^30^. $A$ was calculated to be 4.17 × 10^−5^ C cm^−2^. Consequently, we can image the local surface charge density of single MoS_2_ nanosheets by converting the FFT image with a calibration factor (Fig. 2h). The surface charge density of MoS_2_ is highly related to its vertical charge transport ability, providing a promising tool for uncovering the underlying mechanisms of the electrocatalytic activity of MoS_2_ with various thicknesses.

### Imaging the electrocatalytic activity of monolayer MoS_2_

To image the electrocatalytic activity of monolayer MoS_2_, we introduced an outer-sphere redox mediator, [Ru(NH_3_)_6_]^3+/2+^, which does not undergo physical interactions with the electrode surface. The electrochemical conversion of chemical species between oxidized and reduced states causes variations in the refractive index, which can be detected by our setup. We swept the electrode potential over a monolayer of MoS_2_ between 0 V and −0.4 V (vs. Ag/AgCl) slowly in 0.25 M phosphate buffer containing 10 mM [Ru(NH_3_)_6_]Cl_3_ (Supplementary Fig. 5). The plasmonic images over time and the electrochemical current were recorded simultaneously. Both the conventional cyclic voltammogram and plasmonic intensity of the 1-octadecanethiol-coated gold electrode showed little response, indicating the successful blocking of the potential charging effect of the gold film (Supplementary Fig. 6). This plasmonic imaging technique is sensitive to both surface charging and surface reactions, as discussed above. To differentiate the two kinds of electrochemical responses of a single monolayer MoS_2_, we subtracted the image sequence acquired in phosphate buffer without a redox mediator from the image sequence measured in phosphate buffer with a redox mediator (Supplementary Figs. 7–8). After this subtraction, the obtained plasmonic signal of the redox reaction on monolayer MoS_2_ only reflected the concentration difference between oxidized and reduced molecules. By following the quantitative relationship between the plasmonic signal and the concentration change of the redox species (Supplementary Fig. 9), the subtracted image intensity was converted numerically to the ion concentration ratio of [Ru(NH_3_)_6_]^2+^/[Ru(NH_3_)_6_]^3+^, which enabled us to map the potential-dependent concentration variations of [Ru(NH_3_)_6_]^2+^ ions.

**Fig. 3:**
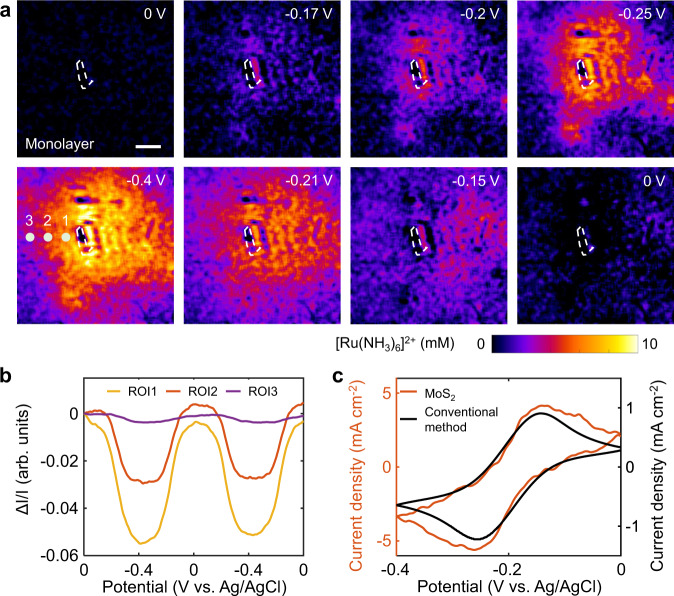
Images of the electrochemical reaction on monolayer MoS_2_. **a** Snapshots of plasmonic images of monolayer MoS_2_ at different potentials, showing the spatial distribution of the concentration of [Ru(NH_3_)_6_]^2+^ ions. The electrolyte was 0.25 M phosphate buffer containing 10 mM [Ru(NH_3_)_6_]Cl_3_. The scale bar represents 15 μm. **b** Plasmonic intensity curves of three ROIs adjacent to monolayer MoS_2_ vs. potential during the potential sweeping. The locations of these ROIs are indicated by colored dots in **a**. **c** Cyclic voltammogram of monolayer MoS_2_ calculated from the plasmonic image sequences (orange line). The cyclic voltammogram measured by the conventional electrochemical method (black line) is shown for comparison.

Figure 3a presents several snapshots of plasmonic images at different potentials, revealing the spatial distribution of the concentration of [Ru(NH_3_)_6_]^2+^ ions (also in Supplementary Movie 1). As the electrode potential decreased, the reduction of [Ru(NH_3_)_6_]^3+^ produced [Ru(NH_3_)_6_]^2+^ molecules, which diffused outward from monolayer MoS_2_ to the surrounding solution. Consequently, the concentration of [Ru(NH_3_)_6_]^2+^ ions increased, and the image contrast gradually appeared around monolayer MoS_2_. We observed the maximum image contrast at −0.4 V, corresponding to the maximum reduction current. As the potential was scanned to 0 V, the image contrast gradually decreased due to the reversible oxidation of [Ru(NH_3_)_6_]^2+^ to [Ru(NH_3_)_6_]^3+^ (Fig. 3a) and finally returned to its original value.

We plotted three regions of interest (ROIs) adjacent to the monolayer MoS_2_ and observed a potential-dependent variation in the plasmonic intensity (Fig. 3b). As the ROI moved away from monolayer MoS_2_, the plasmonic response decreased gradually, indicating the diffusion of [Ru(NH_3_)_6_]^2+^ ions. To quantify this diffusion process, we applied a constant potential of −0.3 V, and the images were recorded synchronously. According to the two-dimensional semi-infinite diffusion model, the concentration of [Ru(NH_3_)_6_]^2+^ ions as a function of diffusion distance (*x*) and time (*t*) follows the equation^31,32^:

2 $$c\left(x,\,t\right)=\frac{M}{\sqrt{4\pi {Dt}}}{{\exp }}\left(-\frac{{x}^{2}}{4{Dt}}\right)$$

where *M* is the total mass of the diffusing ions and *D* is the diffusion coefficient. The diffusion coefficient of [Ru(NH_3_)_6_]^2+^ ions was thus determined to be 4.8 × 10^−10^ m^2^ s^−1^ (Supplementary Fig. 10), which is in accord with the values reported in the literature^33,34^. This diffusion coefficient indicated that the reaction current of MoS_2_ was dominantly controlled by the mass transport of [Ru(NH_3_)_6_]^3+^ ions, which allowed us to calculate the reaction current of monolayer MoS_2_ using linear diffusion models of planar electrodes. The electrochemical current of monolayer MoS_2_ can be determined by performing a Laplace transform according to Fick’s law of diffusion (see Supplementary Note 11 for details). As shown in Fig. 3c, the optical CV (orange line) displays the characteristic redox peaks corresponding to the reduction and oxidation of the ruthenium complex, and this result is in good agreement with the conventional CV curve (black line).

### Quantifying the layer-dependent electrocatalytic activity of single MoS_2_ nanosheets

**Fig. 4:**
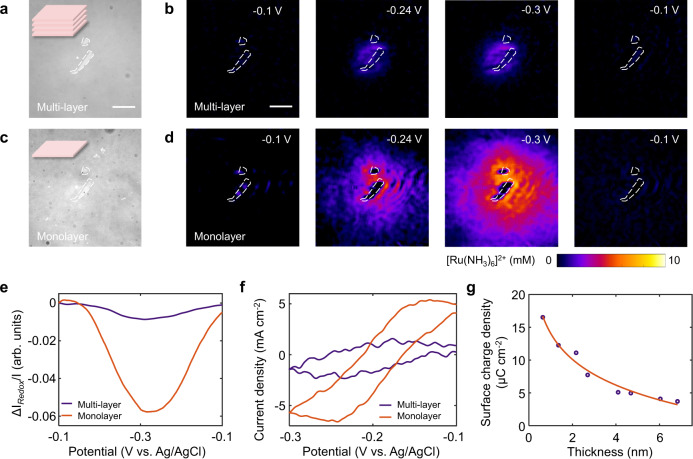
Layer-dependent electrocatalytic activity of single MoS_2_ nanosheets. Optical images and snapshots of plasmonic images of (**a**, **b**) multilayer MoS_2_ and (**c**, **d**) the corresponding surface plasmon-thinned monolayer MoS_2_ during potential sweeping, illustrating the significant difference in the concentration of [Ru(NH_3_)_6_]^2+^ ions. The scale bar represents 20 μm. **e** Plasmonic intensity change (∆*I*/*I*) curves vs. potential of multilayer and monolayer MoS_2_. **f** Cyclic voltammograms of multilayer and monolayer MoS_2_ calculated by the electrochemical imaging. **g** The surface charge density of MoS_2_ dropped quickly as its thickness increased.

The imaging capability of electrocatalytic activity for single MoS_2_ nanosheets enabled us to study the origin of layer-dependent electrocatalytic activity of single MoS_2_ nanosheets, which has been a long-term goal for the community. To unveil the relationship between the layer number and the electrocatalytic activity of MoS_2_, we performed subsequent experiments over multilayer MoS_2_ and its monolayer counterpart fabricated by our surface plasmon etching method^26^. The lateral size of the MoS_2_ nanosheet before and after surface plasmon etching remains unchanged, allowing for the quantification of layer-dependent electrocatalytic activity (Fig. 4a and c). The 1.06 nm height of the nanosheet and the Raman peak shifts indicate that MoS_2_ had been etched into the monolayer (Supplementary Fig. 11). We therefore conducted electrochemical tests using the above MoS_2_ samples. Two movies of the electrocatalytic reaction of MoS_2_ are presented in the Supplementary Movie 2. Figure 4b and c show several snapshots from the movies at different potentials. Surprisingly, we observed a higher image contrast for monolayer MoS_2_, which had a larger diffusion area of [Ru(NH_3_)_6_]^2+^, than for multilayer MoS_2_, implying that monolayer MoS_2_ was more active in accelerating the local electrocatalytic reactions. The change in the plasmonic intensity of multilayer MoS_2_ was observably lower than that of the monolayer sample (Fig. 4e). Figure 4f displays the CVs of multilayer and monolayer MoS_2_ determined from the plasmonic images, and the electrochemical current of monolayer MoS_2_ was higher than that of multilayer MoS_2_, indicating that thinner nanosheets possessed better electrocatalytic activity. More importantly, we also observed that the voltammetric half-wave potential (*E*_1/2_) shifted towards more positive potentials as the thickness of MoS_2_ decreased (Supplementary Fig. 12), further confirming that charge transfer was accelerated as the number of layers was decreased. When we mapped the surface charge density of these single MoS_2_ nanosheets with potential modulation (Supplementary Figs. 13–14), the surface charge density of the MoS_2_ nanosheet decayed rapidly as its thickness increased (Fig. 4g), which further reveals the layer-dependent electrocatalytic activity of MoS_2_. The thicker MoS_2_ has lower Schottky barrier height than the thinner one, leading to a better electron transport ability in thicker MoS_2_ at the Au-MoS_2_ interface (Supplementary Fig. 15). A contrary trend in Fig. 4g indicates that the charge transfer between MoS_2_ and Au contact does not affect the layer-dependent surface charge density of MoS_2_.

## Discussion

**Fig. 5:**
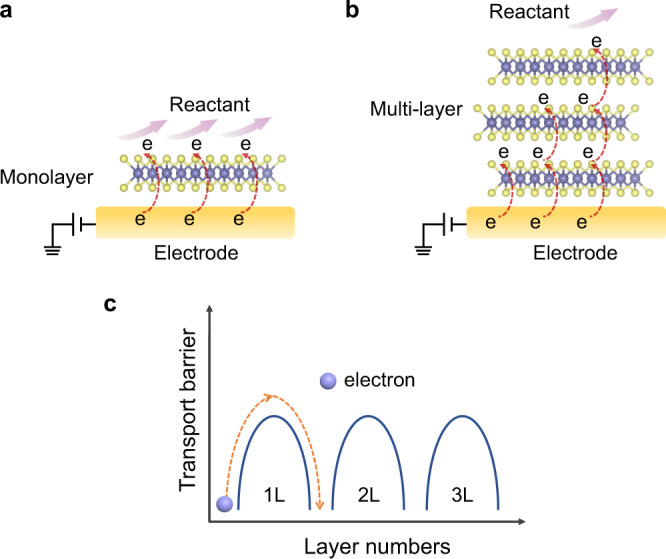
Layer-dependent electron transfer mechanism of MoS_2_. **a**, **b** Schematic illustration depicting electron transfer pathways in the vertical direction of monolayer and multilayer MoS_2_ during the electrochemical reaction. **c** Electrons need to cross more transport barriers with increasing numbers of layers.

In electrocatalysis with MoS_2_, charge transfer from the electrode to the catalytic active sites involves vertical charge transport among layers. Theoretical studies have suggested that the weak van der Waals bonding between the MoS_2_ layers could generate an energy barrier for the interlayer tunneling of the charges^35,36^. Therefore, the charges in a thicker MoS_2_ nanosheet must overcome a higher energy barrier during interlayer tunneling, leading to a lower surface charge density and a lower catalytic activity (Fig. 5). The semiconductor-solution interface dominates the performance of semiconductor electrocatalysis^37^. The accumulation of surface charge could make the semiconductor catalyst conductive, facilitating charge transfer across the interface^19,38^.

Our results demonstrate that a thinner MoS_2_ nanosheet can accumulate charge with a higher concentration to achieve a conductivity that is higher than that of a thicker nanosheet. Consequently, both the capability of charge interlayer tunneling and the conductivity modulated by surface charge density contributed to the better catalytic efficiency of the thinner MoS_2_. Unlike the existing methods for electrochemical measurement of single 2D material nanosheets with nanofabrication^19^, our method is easy and facile. Microelectrode-based measurements have provided insights into the electrochemical reaction of 2D MoS_2_ nanosheets^39^, but these methods are limited by low throughput and temporal resolution and cannot acquire real-time electrochemical information, such as can be obtained through diffusion dynamics. Our approach was based on wide-field imaging without the use of a scanning microelectrode, which enabled a fast imaging speed and avoided the possible disturbance of the sample by the microelectrode. More importantly, we can directly visualize the heterogeneous distribution of surface charge, which is associated with the electrocatalytic performance of MoS_2_.

We also demonstrated the use of this technique in studying the electrocatalytic hydrogen evolution reaction (HER) on single MoS_2_ nanosheets (see Supplementary Note 16 and Supplementary Fig. 16 for details). The generated hydrogen molecule leads to the decrease in the local refractive index around MoS_2_, which is reflected in the change of plasmonic image contrast. Supplementary Fig. 16b displays snapshots of differential plasmonic images during cathodic potential sweeping, where the image contrast decreases as the potential decreases, and achieves a maximum at the lowest potential. The electrochemical current was converted from the plasmonic signal change, revealing the remarkable hydrogen evolution for MoS_2_ around −0.4 V (Supplementary Fig. 16c, d). Moreover, to demonstrate the generality of this imaging method, we probed the electrocatalytic activity of other 2D materials, including graphene, WS_2_ and MoSe_2_, which exhibited similar layer-dependent electrochemical behaviors (Supplementary Fig. 17). All these results suggest that interlayer charge transfer plays a considerable role in the electrocatalysis of 2D materials. The results obtained could also be used to explain the high electrocatalytic activities of nanostructured semiconductor catalysts^40^ and TMDs^5^, since these materials can be made conductive by making them thinner.

The spatial resolution of our plasmonic imaging technique is about 250 nm due to the optical diffraction limit, which is lower than that of probe-scanning based imaging techniques for visualizing active site distributions of heterogeneous electrocatalysts^41–43^. Despite of the resolution limit, it is sufficient to probe the surface charge density as a function of the thickness of MoS_2_ flakes. Future integration with other high-resolution characterization technologies will allow multifunctional monitoring of a nanostructured electrocatalyst toward establishing structure-activity relationships.

In summary, we have demonstrated a plasmonic imaging method that enables the visualization of the surface charge density and electrocatalytic activity of single 2D electrocatalyst nanosheets in situ. This imaging capability allowed us to study the layer-dependent electrocatalytic activity of MoS_2_ and further correlate the layer-dependent electrocatalytic activity with the surface charge density of single MoS_2_ nanosheets. Our findings provide additional insights into understanding the electrocatalytic mechanisms of TMDs, and this optical imaging technique can serve as a transformative platform for investigating the microscopic interfacial electrochemical processes of 2D electrocatalysts.

## Methods

### Instrumentation

The plasmonic imaging setup was built on an inverted total internal reflection microscope (ECLIPSE Ti-E Series, Nikon Instruments Inc.) equipped with a high numerical aperture (NA = 1.49) oil-emersion objective (60×). A beam of *p*-polarized light from a 680 nm superluminescent diode (SUPERLUM, Ireland) was directed onto the gold film mounted on the objective to excite surface plasmon polaritons, and the reflected light was collected with the same objective and directed to an sCMOS (ORCA-Flash4.0 C11440, Hamamatsu Photonics K.K.), or CCD camera (Pike F-032B, Allied Vision Technologies GmbH) for imaging. The gold chips were prepared by evaporating 2 nm chromium as an adhesion layer followed by a 47 nm gold layer on BK-7 glass coverslips. The gold films were immersed in 10 mM 1-octadecanethiol dissolved in ethanol for 48 h, rinsed with ethanol and water sequentially, and dried with nitrogen gas prior to use.

### Sample preparation

Bulk MoS_2_ single crystals were purchased from Nanjing MKNANO Tech. Co., Ltd. 1-Octadecanethiol and [Ru(NH_3_)_6_]Cl_3_ were purchased from Sigma Aldrich. The MoS_2_ monolayers were fabricated by a surface plasmon polariton-driven etching approach recently developed by our group. A laser diode with a wavelength of 660 nm (OBIS LX, Coherent Inc.) was adopted as the light source to induce layer thinning of the MoS_2_ nanosheets. The power density of red light was generally set at 3 mW·mm^−2^. The MoS_2_ multilayers were mechanically exfoliated from bulk MoS_2_ single crystals and transferred onto 1-octadecanethiol-modified gold films. The MoS_2_ multilayers were directly converted into corresponding monolayers under surface plasmon-driven etching in deionized water^26^.

### Electrochemical measurements

The MoS_2_ nanosheet-deposited gold film was employed as the working electrode (a platinum wire as the counter electrode and Ag/AgCl as the reference electrode) to construct a three-electrode electrochemical cell. A polydimethylsiloxane (PDMS) electrochemical cell was placed on top of the gold film. For the charging experiment, the electrode potential was applied and controlled using an electrochemical workstation (CorrTest-CS5, Wuhan Corrtest Instrument Corp. Ltd) coupled with an external waveform function generator (33500B Trueform, Keysight Technologies Inc.). A data acquisition card (DAQ USB-6250, National Instruments Corp.) was utilized to synchronize the electrochemical measurements and the CCD camera (100 frames/s). In the charging experiment, cyclic voltammetry was conducted by sweeping the potential between 0 V and −0.4 V (vs. Ag/AgCl) in 260 mM PB solution. A sinusoidal potential modulation amplitude of 100 mV and frequency of 20 Hz were also applied to the sample. In the redox experiment, CV was carried out on an electrochemical workstation (CHI660E, CH Instruments Inc.), and the sequence of plasmonic images was recorded by a sCOMS camera at 200 frames/s. CV was conducted in 260 mM PB (charging) and 250 mM PB solutions containing 10 mM [Ru(NH_3_)_6_]Cl_3_ (redox). In the layer-dependent experiments, we first performed electrochemical tests with the mechanically exfoliated MoS_2_ multilayers, then etched this sample into corresponding monolayers in situ with planar surface plasmons^26^, and finally, we performed the same electrochemical tests again with the obtained MoS_2_ monolayers.

### Characterization

Atomic force microscopy (AFM) was performed using a Bruker Dimension Icon system in tapping mode. A Raman spectroscope (LabRAM Nano, HORIBA France SAS) with a laser excitation wavelength of 532 nm was used to obtain the Raman spectra and photoluminescence spectrum of the MoS_2_ nanosheets.

### Data processing

We employed MATLAB and ImageJ with self-developed codes to process images in this work. In the charging experiment, fast Fourier transform (FFT) was performed on each pixel of the recorded images in the time domain.

## Supplementary information

Supplementary Information

Description of Additional Supplementary Files

Supplementary Movie 1

Supplementary Movie 2

## Data Availability

All data are available within the Article and Supplementary Files. Source data are provided as a Source Data file. Source data are provided with this paper.

## Source data

Source Data

## Code availability

The code for the image process in this paper is available in Github: https://github.com/XiaonaZhao/Intensity2Current.
