# Supplementary material for: Plasmonic imaging of the layer-dependent electrocatalytic activity of two-dimensional catalysts
Source: Nat Commun. 2022 Dec 22;13:7869. doi: 10.1038/s41467-022-35633-3 (PMC9780338; doi:10.1038/s41467-022-35633-3)
Supplement: Supplementary file 1 — Supplementary Information [file 41467_2022_35633_MOESM1_ESM.pdf]

Supplementary Information for

Plasmonic Imaging of the Layer-dependent Electrocatalytic Activity of Two-dimensional Catalysts

Xiaona Zhao[1], Xiao-Li Zhou[1,2*], Si-Yu Yang[1], Yuan Min[1], Jie-Jie Chen[1], and Xian-Wei Liu[1,3*]

[1]Chinese Academy of Sciences Key Laboratory of Urban Pollutant Conversion, Department of Environmental Science and Engineering, University of Science and Technology of China, Hefei, 230026, China

[2]School of Environmental and Biological Engineering, Nanjing University of Science and Technology, Nanjing 210094, China

[3]Department of Applied Chemistry, University of Science and Technology of China, Hefei, 230026, China

*Correspondence to: xlzhou@njust.edu.cn (XL Zhou) or xianweiliu@ustc.edu.cn (XW Liu)

Supplementary Notes

1. Layer thinning of MoS_2 for the charging experiment 3

2. Plasmonic response to charge density change of MoS_2 4

3. Image processing method 6

4. Equivalent circuits for the charging experiment 8

5. Layer thinning of MoS_2 for the redox reaction of the $[Ru(NH_3)_6]^{3+}$ complex 11

6. Cyclic voltammetry of the bare Au and 1-octadecanethiol-modified Au electrodes 12

7. Extraction of faradaic process by charging process subtraction 13

8. Quantification of the noise level 14

9. Calibrating the concentration of redox species from the plasmonic image intensity 16

10. Determining the diffusion coefficient of $[Ru(NH_3)_6]^{2+}$ ions 19

11. Conversion of the plasmonic intensity of single MoS_2 nanosheets to electrochemical current 20

12. Characterizations of MoS_2 for layer-dependent catalytic experiments 21

13. Catalytic kinetic analysis of MoS_2 with different thicknesses 22

14. Mapping the surface charge density of single MoS_2 nanosheets with various thicknesses 24

15. Au-MoS_2 Schottky contacts 27

16. Imaging the hydrogen evolution reaction (HER) at single MoS_2 nanosheets. 28

17. Layer-dependent electrocatalytic activity of other two-dimensional materials 30

1. Layer thinning of MoS_2 for the charging experiment

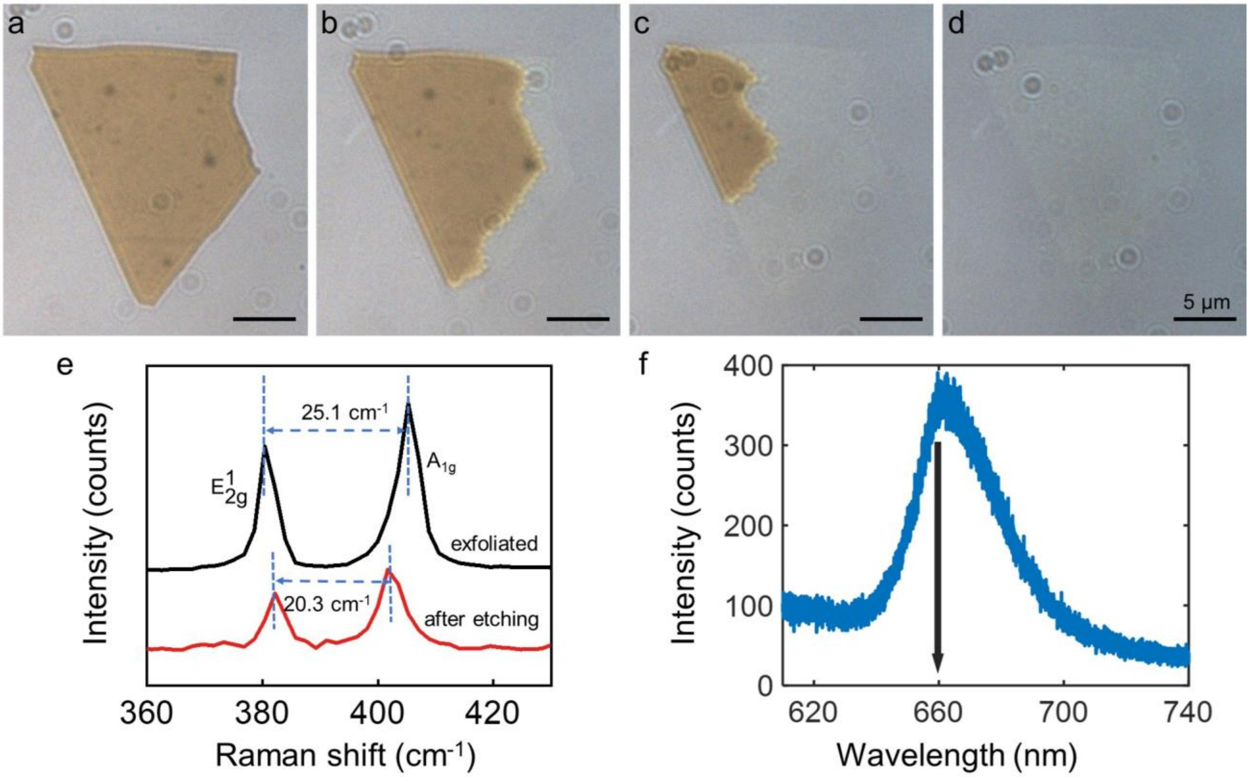

Supplementary Fig. 1 Monolayer MoS_2 obtained via the surface plasmon-driven etching strategy. (a-d) Time-dependent optical images showing the etching process of a MoS_2 nanosheet. (e) Raman spectra of the MoS_2 nanosheet after etching and pristine exfoliated nanosheet for comparison. The MoS_2 nanosheet after etching shows an obviously smaller peak difference between the E_{2g}^{1} and A_{1g} peaks (20.3 cm^{-1}) than those of the pristine MoS_2 nanosheet (25.1 cm^{-1}), suggesting the monolayer structure of MoS_2. (f) Photoluminescence (PL) spectrum of the MoS_2 nanosheet after etching.

2. Plasmonic response to charge density change of MoS_2

In the surface plasmon resonance (SPR) imaging setup, a beam of *p*-polarized monochromatic light passes through a high numerical aperture objective to illuminate the gold-coated coverslip. When the incident angle is modulated, a sharp decrease in reflectivity can be monitored with a camera. The angle at the reflectivity minimum is referred to as the resonance angle. The resonance angle (θ_{SPR}) is given by

$$\sin(\theta_{SPR}) = \sqrt{\frac{\varepsilon_1 \varepsilon_m}{(\varepsilon_1 + \varepsilon_m)\varepsilon_2}}$$

where ε_1, ε_2, and ε_m are the dielectric constants of the electrolyte, objective, and metal film, respectively. Previous research has demonstrated that the refractive index of monolayer MoS_2 decreases with increasing electron density[1, 2]. When a negative potential is applied to MoS_2, electrons flow into MoS_2, resulting in an increase in the electron density. This phenomenon causes ε_1 and θ_{SPR} to decrease according to the above Equation[3, 4, 5].

The decrease of θ_{SPR} led to a decrease in the plasmonic intensity. The plasmonic intensity change ($\Delta I/I$) is converted from the resonance angle shift ($\Delta\theta_{SPR}$)

$$\Delta I/I = \beta \times \Delta\theta_{SPR}$$

where β is set by the parameter of the oil-emersion objective (60x) and the laser power of 0.2 mW/mm^2. The charge density change can be expressed in terms of the dielectric constant change

$$\Delta q = -\frac{e d_m n_e}{\varepsilon_m - 1}\Delta\varepsilon_m$$

where d_m is the thickness of the metal film and n_e is the electron density. Combining the former three equations, we have

$$\Delta q = -\frac{\beta e d_m n_e \varepsilon_2 (\varepsilon_1 + \varepsilon_m)^2 \sin(2\theta_{SPR})}{{\varepsilon_1}^2(\varepsilon_m - 1)}\Delta I/I$$

This equation can be abbreviated as $\Delta q = A\, \Delta I/I$, where A is a calibration factor. This equation reveals that the potential modulation changes surface charge density of MoS_2 and thus alters the dielectric constant of MoS_2, which is externally manifested as the variation of plasmonic intensity.

Here we sought to simulate the SPR reflectivity profile of monolayer MoS_2 at different applied potentials for the optical multilayer interface[6]. The Transfer Matrix method is based on the continuity conditions for the electric field across boundaries from one medium to the next,

according to Maxwell's equations. In our experimental system, the reflections of light in the multiple interfaces are partially transmitted and reflected. The overall reflection of a layer structure is the sum of an infinite number of reflections (Supplementary Fig. 2a). The refractive index of MoS_2 under different potential was acquired from the literature[7]. The increased electron density of MoS_2 decreases its refractive index. In Supplementary Fig. 2b and c, the application of a negative potential on the MoS_2 shifts the θ_{SPR} towards lower incidence angles. A convenient way to determine the potential-dependent θ_{SPR} is to fix the incident angle near the resonant angle (71.5◦, dashed line in Supplementary Fig. 2c) and measure the plasmonic intensity change in the reflected light. As a result, the plasmonic intensity of monolayer MoS_2 decreases with the increase in electron density.

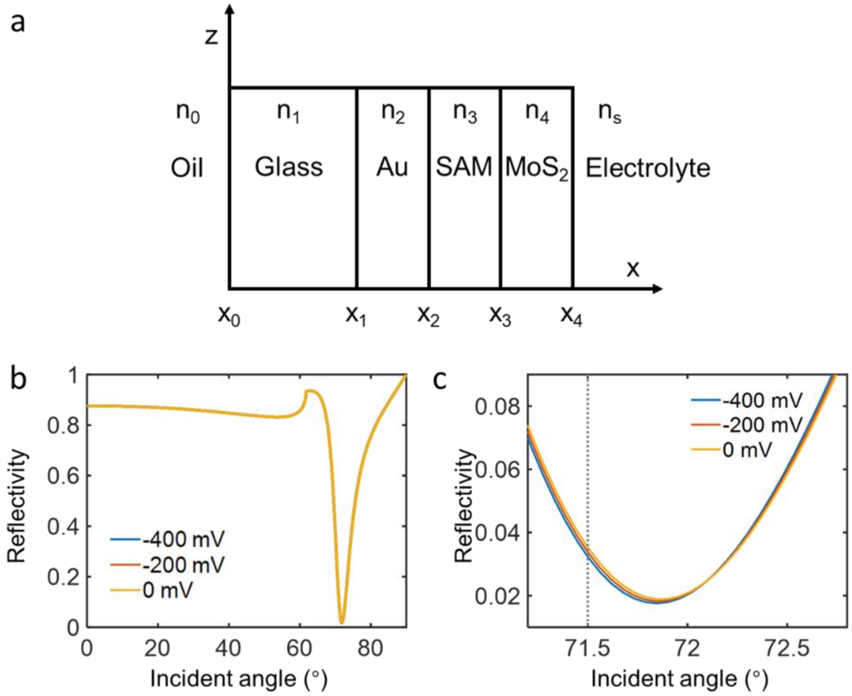

Supplementary Fig. 2 Plasmonic response to charge density change of MoS_2. (a) The layer structure of optical imaging platform. (b, c) The simulated SPR reflectivity profile of monolayer MoS_2 at different applied potentials.

3. Image processing method

In the charging experiments, the electrochemical recording was synchronized with the camera by adding a timeline into the image sequences using a data acquisition card (DAQ, USB-6250, National Instruments). We subtracted the first image of each sequence (Supplementary Fig. 3a-b). With the timestamps, we selected one of the $\frac{\pi}{2}$ points in the sine wave of the voltage timeline, as shown in Supplementary Fig. 3c, and extracted 2048 frames of the subtracted image sequence from the selected point. The extracted image stack was transformed pixel by pixel into one amplitude image through fast Fourier transform (FFT) and one phase image with the comparison of the voltage wave (Supplementary Fig. 3d). The surface charge density of the sample was displayed by the amplitude image, which was calibrated with the optical response constant from a linear voltage scan experiment (Supplementary Fig. 3e-f).

In the redox experiments, timestamps were added to the recorded image sequences, and the image of the initial potential was subtracted (Supplementary Fig. 3g). To remove the temporal noise efficiently, the subtracted image stack was filtered by a Chebyshev window-based FIR filter, where bands of frequencies lower than 0.065 Hz and higher than 15 Hz were cut off. To decrease the spatial noise, the filtered images were spatially smoothed with a 15×15 pixel (3.75×3.75 μm) filter kernel. In the sampling area, local response profiles were plotted as traces of image intensity changes at selected regions of interest (ROIs). The optical response was examined and displayed by subtracting the image of the initial potential (Supplementary Fig. 3h). After that, the plasmonic signal was transformed into the concentration of $[Ru(NH_3)_6]^{2+}$ ions with our previous calibration (Supplementary Fig. 3i). According to the semi-infinite diffusion theory and Laplace transform (Supplementary Fig. 3j), the optical response was finally transferred into the localized current density.

All analyses above were carried out by custom-written MATLAB code, which is available upon request.

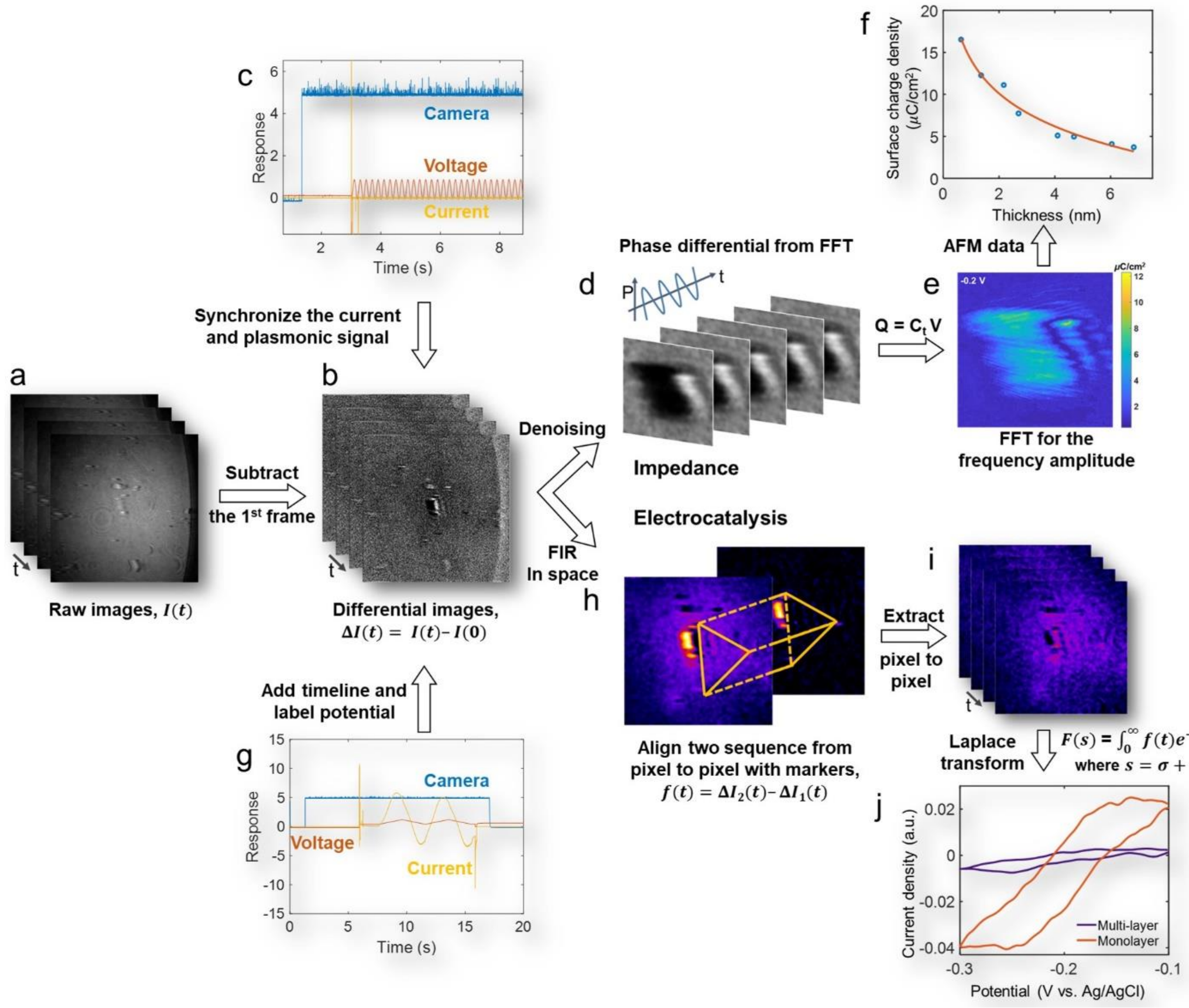

Supplementary Fig. 3 Workflow of the image processing in this work. Workflow of the image processing for measuring the surface charge density (a-b, c-f) and the electrocatalytic activity of single MoS_2 nanosheets (a-b, g-j). Commonalities: (a) Raw images from CCD cameras. (b) Differential images after subtracting the 1st frame of the image sequence. Surface charge density: (c) adding timelines to all the signals obtained from the electrochemical workstation and cameras, (d) denoising and extracting the phase and amplitude of the MoS_2 nanosheet and bare gold electrode, (e) reconstructing the charge distribution image with the potential, and (f) plotting the curve of the charge density vs. thickness from AFM data. Electrocatalysis: (g) adding the timeline, (h) extracting two image sequences pixel to pixel with the corresponding potential, (i) calibrating the concentration of redox species from the plasmonic image intensity, and (j) obtaining the conventional cyclic voltammogram from ion concentrations with Laplace transform.

4. Equivalent circuits for the charging experiment

Considering the absence of redox species in the charging experiment, the electrochemical system was dominated by the interfacial capacitances and resistance in series. As shown in the equivalent circuit (Supplementary Fig. 4a), the interfacial capacitance in the monolayer MoS_2 area consists of electrical double layer capacitance (C_{EDL}) and quantum capacitance (C_Q) in series: $\frac{1}{C} = \frac{1}{C_{EDL}} + \frac{1}{C_Q}$. The C_{EDL} is mainly dependent on the electrolyte concentration and ion species. In 0.26 M phosphate buffer, the C_{EDL} was approximately 25 μF cm^{-2} [8]. The C_Q of monolayer MoS_2 was calculated to be 5 μF cm^{-2} by the following equation[9]:

$$C_Q = e^2 g_{2D}\left[1 + \frac{exp\left(\frac{E_G}{2k_BT}\right)}{2\cosh\left(\frac{eV_g}{k_BT}\right)}\right]$$

where g_{2D} is the band-edge DOS, and E_G is 1.9 eV for the monolayer MoS_2. V_g is the gate potential, -0.2 V. More detailed calculation for quantum capacitance was provided in Supplementary Note 14.

Traditional electrical methods measure the average signal of the whole working electrode. In our work, MoS_2 nanosheets were distributed sparsely on the working electrode. The total capacitance of the whole electrode interface was mainly dominated by electrical double layer capacitance, since the electrical signal of the MoS_2 nanosheets was negligible due to the low coverage of MoS_2 nanosheets on the electrode. In contrast, the imaging capability of our plasmonic technique allows for imaging of local interfacial capacitance. For the plasmonic imaging, the current or charge response to the potential modulation between the Au/SAM/MoS_2/electrolyte region and nearby Au/SAM/electrolyte region were totally different. The plasmonic intensity ($\Delta I/I$) of monolayer MoS_2 shows a periodic oscillation with the applied potential while the plasmonic intensity of the Au electrode remained constant (Supplementary Fig. 4b). A phase shift takes place between the waveforms of plasmonic intensity and the applied potential due to the existence of solution resistance (R_S). The relationship between the plasmonic intensity change ($\Delta I/I$) and the surface charge density change (Δq) of MoS_2 is $\Delta q = A\Delta I/I$, while the charge density change comes from the variation of AC current over time, which is given by $\Delta q = i\Delta t$. The plasmonic signal is converted to the localized electrical signal with the surface charge density of MoS_2, which takes the form of $\Delta q = A\Delta I/I = i\Delta t$. In impedance or AC analysis[10, 11], the phase shift indicates that the

AC current lags behind AC potential, $V = \left(\frac{1}{j2\pi f C_Q} + \frac{1}{j2\pi f C_{EDL}} + R_s\right) \times i$, by 0° to 90°, where j is the imaginary unit and f is the frequency of potential. In the absence of the solution resistance, the phase shift between potential and current is 90° and is not dependent on the modulation frequency[3, 12]. Herein, the charge density change is the function of the modulation potential, $\Delta q = \frac{2\pi f C_{EDL} C_Q}{R_S 2\pi f C_{EDL} C_Q - j(C_{EDL}+C_Q)} \Delta V$, and the phase shift between Δq and potential (ΔV) is highly dependent on the frequency of potential due to the presence of the solution resistance. The intensity and phase shift suggested that the plasmonic imaging can directly visualize the heterogeneous distribution of local interfacial capacitance of the two regions. Moreover, the current response of the whole working electrode to a potential modulation was also recorded by the electrochemical workstation (Supplementary Fig. 4c). The phase shifts measured from the plasmonic and electrical signals were not synchronized, for the resistance and capacitance of other components existing in the full electrochemical system.

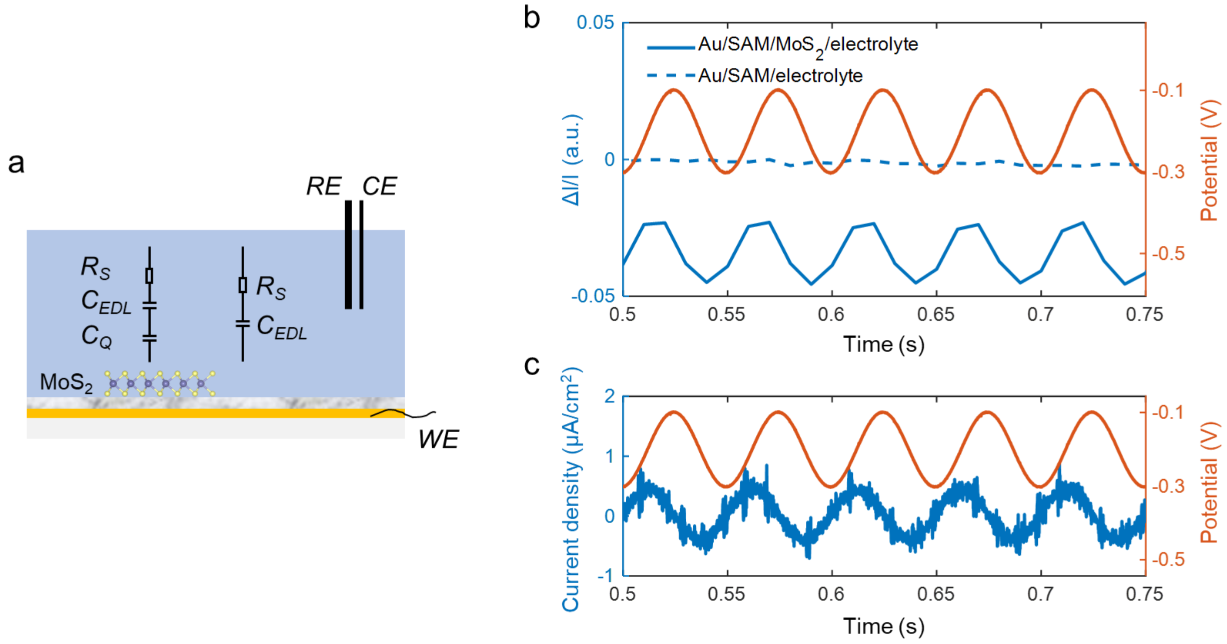

Supplementary Fig. 4 The interfacial equivalent circuits clarify the plasmonic and electric response to the potential modulation. (a) Schematic illustration showing the equivalent circuits of the monolayer MoS_2 area (left) and Au region (right), where R_S, C_{EDL}, and C_Q represent the solution resistance, double layer capacitance, and quantum capacitance of monolayer MoS_2, respectively. (b) The plasmonic intensity responses of the Au/SAM/MoS_2/electrolyte and

Au/SAM/electrolyte region to the potential modulation. (c) The current response to the applied potential measured by the electrochemical workstation.

The surface charge density of single MoS_2 nanosheet is related to local interfacial capacitance per unit area (c), $\Delta q = c\Delta V$. Accordingly, we need to obtain the interfacial capacitance of the MoS_2 region instead of the total capacitance of the whole working electrode. Previous literatures have also adopted the series-connected capacitors of C_Q and C_{EDL} to represent the interfacial capacitance of the semiconductor–electrolyte interface ($\frac{1}{C} = \frac{1}{C_{EDL}} + \frac{1}{C_Q}$)[13, 14]. Therefore, it is reasonable to use the C_Q for the final capacitance of the MoS_2–electrolyte interface, regardless of the right loop describing the Au/SAM/electrolyte region.

5. Layer thinning of MoS_2 for the redox reaction of the $[Ru(NH_3)_6]^{3+}$ complex

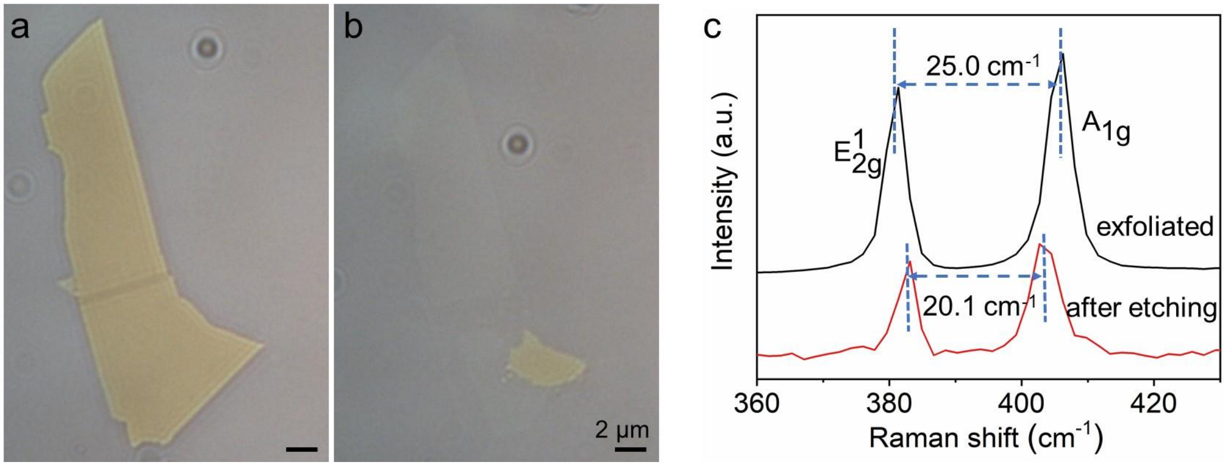

Supplementary Fig. 5 Characterization of the monolayer MoS_2 used in the catalytic experiments. (a) Optical image of a pristine exfoliated MoS_2 nanosheet. (b) Optical image of monolayer MoS_2 after etching. (c) Raman spectra of the MoS_2 nanosheet after etching and of pristine exfoliated nanosheet for comparison. After etching, the MoS_2 nanosheet showed a smaller peak difference between the E^1_{2g} and A_{1g} peaks (20.1 cm^{-1}) than those of the pristine MoS_2 nanosheet (25.0 cm^{-1}), suggesting the monolayer structure of MoS_2.

6. Cyclic voltammetry of the bare Au and 1-octadecanethiol-modified Au electrodes

The Au electrode was passivated by a self-assembled monolayer (SAM) of 1-octadecanethiol molecules to hinder electron transfer from the gold electrode to the redox molecules. To demonstrate the effectiveness of blocking, we simultaneously studied the redox reaction of the $Ru(NH_3)_6^{3+}$ complex with conventional CV and plasmonic imaging methods. As shown in Supplementary Fig. 6a, the cyclic voltammogram of $[Ru(NH_3)_6]^{3+/2+}$ for the bare Au electrode shows a pair of characteristic reversible redox peaks. The simultaneously measured plasmonic response vs. potential presents a sigmoidal change as the redox couple was switched between the oxidized and reduced states (Supplementary Fig. 6b). Both the cyclic voltammogram and plasmonic response of the 1-octadecanethiol-modified Au electrode showed little change during potential cycling, demonstrating that the electron transfer pathway of the gold electrode was successfully blocked.

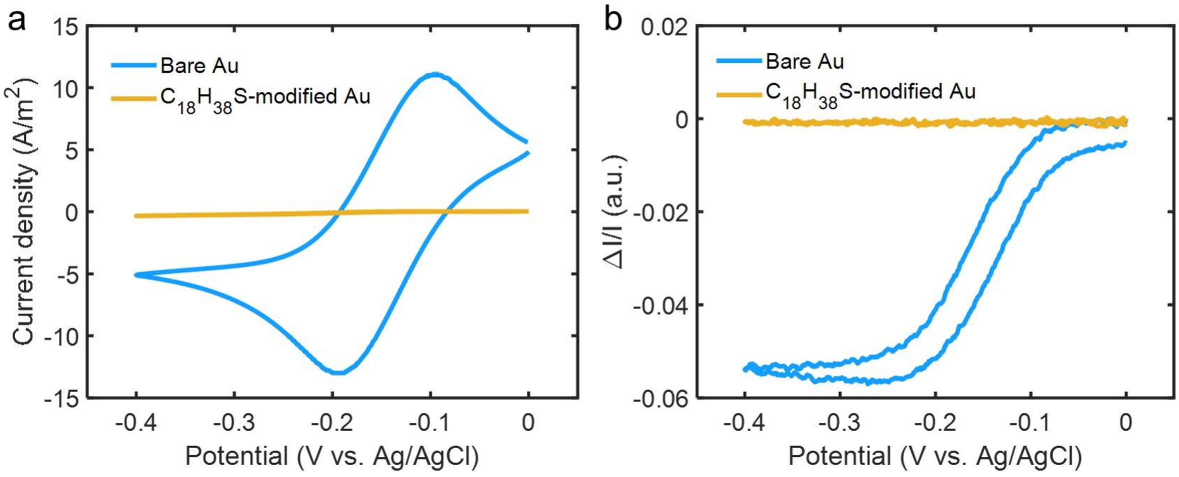

Supplementary Fig. 6 Cyclic voltammetry of the bare Au and 1-octadecanethiol-modified Au electrodes. (a) Traditional cyclic voltammograms of the bare Au (blue) and 1-octadecanethiol-modified Au (yellow) electrodes measured by the electrochemical workstation. (b) The simultaneously recorded plasmonic response vs. potential of the bare Au (blue) and 1-octadecanethiol-modified Au (yellow) electrodes. The electrolyte is 0.25 M phosphate buffer containing 10 mM $[Ru(NH_3)_6]Cl_3$, and the scan rate was 100 mV s^{-1}.

7. Extraction of faradaic process by charging process subtraction

During electrochemical cycling in 0.25 M phosphate buffer containing 10 mM $[Ru(NH_3)_6]Cl_3$, the plasmonic images recorded both the signal of charge density variation within monolayer MoS_2 (Supplementary Fig. 7a) and the signal of redox reaction of $[Ru(NH_3)_6]^{3+/2+}$ on monolayer MoS_2 (Supplementary Fig. 7b). To deduct the charging signal, we subtracted the image sequence of the charging experiment from the image sequence of the redox experiment. The results are shown in Supplementary Fig. 7c. After the deduction, the obtained plasmonic signal of the redox reaction on monolayer MoS_2 reflected the concentration difference between oxidized and reduced molecules.

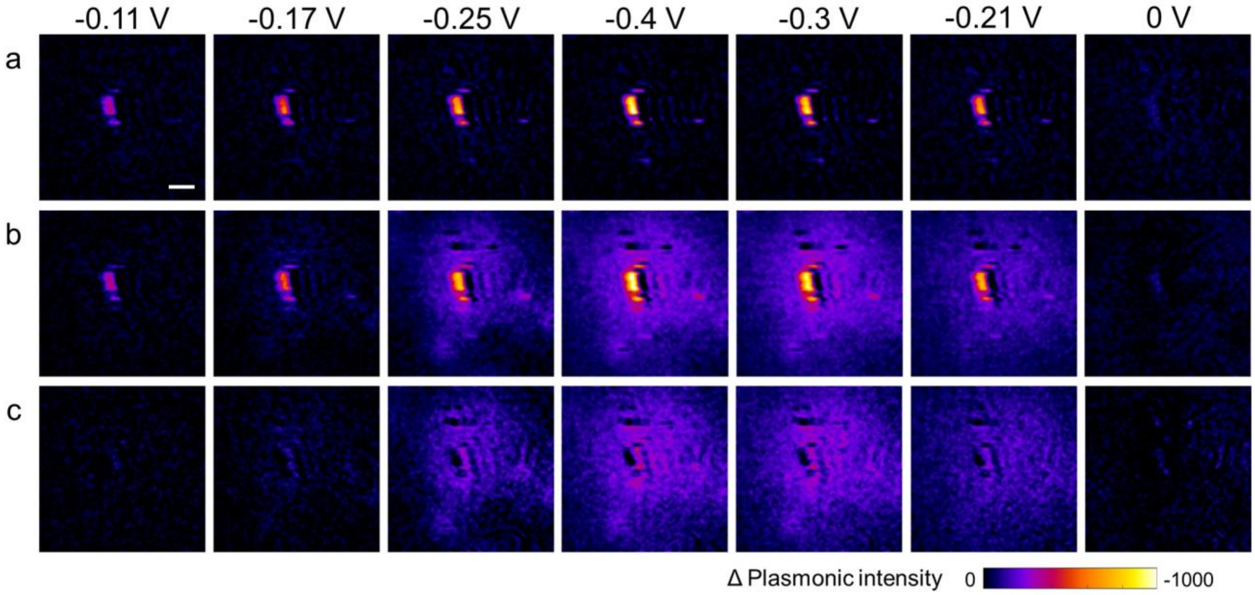

Supplementary Fig. 7 Extraction of faradaic process by charging process subtraction. (a) Snapshots of difference plasmonic images of monolayer MoS_2 in the charging experiment. Scale bar: 15 μm. The electrolyte is 0.26 M phosphate buffer (pH = 6.86). The electrode potential was cycled between 0 V and -0.4 V (vs. Ag/AgCl), and the scan rate was 100 mV s^{-1}. Each of the difference images was obtained by subtracting the first image at 0 V. (b) Snapshots of difference plasmonic images of the same monolayer MoS_2 in the redox experiment. The electrolyte is 0.25 M phosphate buffer containing 10 mM $[Ru(NH_3)_6]Cl_3$. (c) Snapshots of plasmonic images obtained by subtracting the image sequence of the charging experiment from the image sequence of the redox experiment.

8. Quantification of the noise level

We quantified the noise level of the plasmonic response for the electrochemical redox reaction. Along with the potential modulation, the plasmonic response of monolayer MoS_2 in different concentrations of $[Ru(NH_3)_6]Cl_3$ solution had different signal-to-noise ratios (SNRs). The noise originated from the recorded and filtered signals. Estimated with their power spectral density, the SNR was calculated with the following equation[15]:

$$\mathrm{SNR} = \frac{P_{signal}}{P_{noise}} \cong \frac{P_{filtered}}{P_{recorded-filtered}} = \frac{x_f^2(t)}{\left(x_r(t) - x_f(t)\right)^2}$$

Signals recorded and filtered from $[Ru(NH_3)_6]Cl_3$ solutions at different concentrations are presented in Supplementary Fig. 8a.

The SNR results show that the electrolytes with different concentrations of $[Ru(NH_3)_6]Cl_3$ presented a stable plasmonic response to the potential modulation. When the solution contained no $[Ru(NH_3)_6]Cl_3$, the SNR was less than 1 dB, which meant that the plasmonic signal underwent little change during potential modulation (Supplementary Fig. 8b). With increasing concentration, the SNR increased quickly, as shown in Supplementary Fig. 8c-f. The SNR tended to slowly rise when the concentration of $[Ru(NH_3)_6]Cl_3$ was over 10 mM for the saturation of the electrochemical reaction on monolayer MoS_2. Therefore, we used a 10 mM $[Ru(NH_3)_6]^{3+}$ electrolyte as the redox probe in the electrochemical experiments.

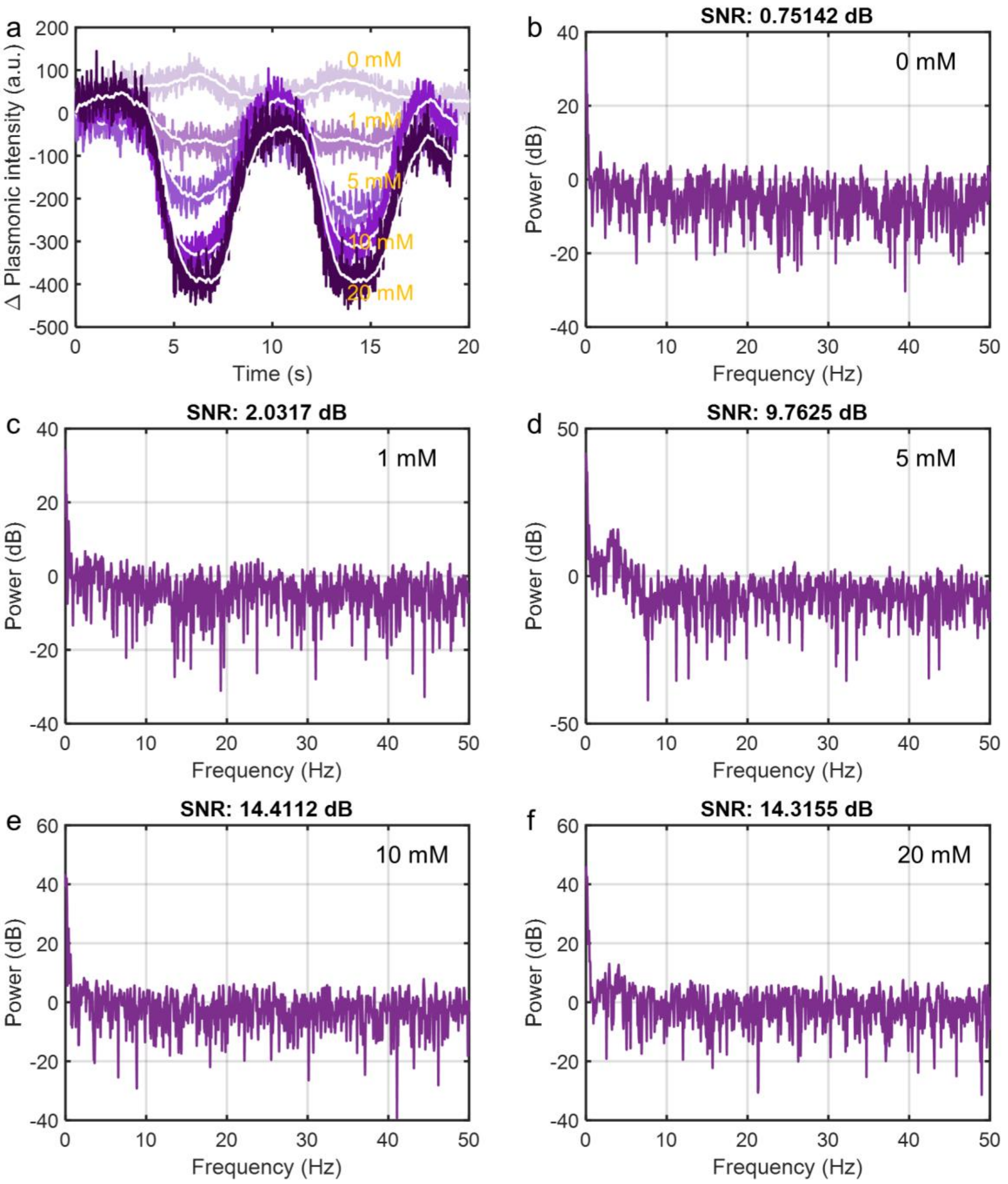

Supplementary Fig. 8 The noise level of the plasmonic signals. (a) The recorded and filtered plasmonic intensity changes of monolayer MoS_2 during potential sweeping in different concentrations of $[Ru(NH_3)_6]Cl_3$ solution. (b-f) The power spectral density spectra and the SNR of plasmonic imaging at different concentrations of $[Ru(NH_3)_6]Cl_3$ solution.

9. Calibrating the concentration of redox species from the plasmonic image intensity

To convert the observed plasmonic signal response into changes in the concentration of $[Ru(NH_3)_6]^{2+}$ ions, we performed electrochemical experiments using the bare Au electrode without modification. Since the bare Au electrode possessed a much higher electrical conductivity and electron transfer rate than monolayer MoS_2, the $[Ru(NH_3)_6]^{3+}$ ions were completely reduced to $[Ru(NH_3)_6]^{2+}$ ions. As shown in Supplementary Fig. 9a (blue), 10 mM $[Ru(NH_3)_6]Cl_3$ was gradually reduced to 10 mM $[Ru(NH_3)_6]Cl_2$ from 0 V to -0.4 V, while the plasmonic intensity decreased by 600 intensity units (IU) (all the plasmonic intensity changes were relative to that at a potential of 0 V).

To acquire the plasmonic intensity shift per unit concentration change of the $[Ru(NH_3)_6]^{2+}$ ions, we performed the same experiment using a commercial prism-based surface plasmon resonance (SPR) system (Bi4500 from Biosensing Instruments, www.biosensingusa.com). The SPR angular shifts per unit concentration change for the reduction of $[Ru(NH_3)_6]^{3+}$ to $[Ru(NH_3)_6]^{2+}$, $B\alpha_R - B\alpha_O$, was determined to be 2.5 mDeg mM^{-1}, and the corresponding plasmonic intensity shift was 120 IU mM^{-1}. Then, we simulated the plasmonic intensity shift for the reduction process of 10 mM $[Ru(NH_3)_6]^{3+}$ ions, according to the Nernst equation and the local plasmonic signal equation[16]:

$$E = E_0 - \frac{RT}{nF} ln\left(\frac{a_R}{a_O}\right)$$

$$\theta_{SPR}(t) = B[\alpha_R c_R(z,t)|_{z=0} + \alpha_O c_O(z,t)|_{z=0}]$$

Since the redox couple is a strong electrolyte, the thermodynamic activity can be replaced by the ion concentration, that is, $a_R = c_R$, $a_O = c_O$. Then, according to the reaction conditions $c_O(z,t)|_{z=0,t=0}$ equal to 10 mM and $c_R(z,t)|_{z=0}$ changing from 0.0001 to 9.9999 mM, the simulated plasmonic intensity change vs. the concentration change of the redox species displays an S-shaped curve (Supplementary Fig. 9a, orange). We observed a one-to-one correspondence between the measured plasmonic intensity change and the simulated plasmonic intensity change determined according to Eq. 2.

To validate the relationship between the concentration ratio of $[Ru(NH_3)_6]^{3+/2+}$ and the measured plasmonic intensity change, we converted the measured current to the plasmonic intensity, or vice versa[17]. Supplementary Fig. 9b (the orange curve) shows the semi-integral of the conventional cyclic voltammogram acquired on an electrochemical workstation, which was in

accord with the measured plasmonic intensity change (the blue curve). Additionally, the current-potential curve converted from the measured plasmonic intensity coincided with the conventional CV measured with an electrochemical workstation (Supplementary Fig. 9c). These two curves were overlayed accurately, indicating that the plasmonic intensity change and the conventional CV could be converted to each other. When the redox reaction was performed on the 1-octadecanethiol-modified gold electrode, the plasmonic intensity change in 10 mM $[Ru(NH_3)_6]Cl_3$ presented a similar plasmonic intensity shift (Supplementary Fig. 9d). This result indicates that an alkanethiol layer could block the electrochemical reaction of the gold substrate without affecting the redox reaction of monolayer MoS_2.

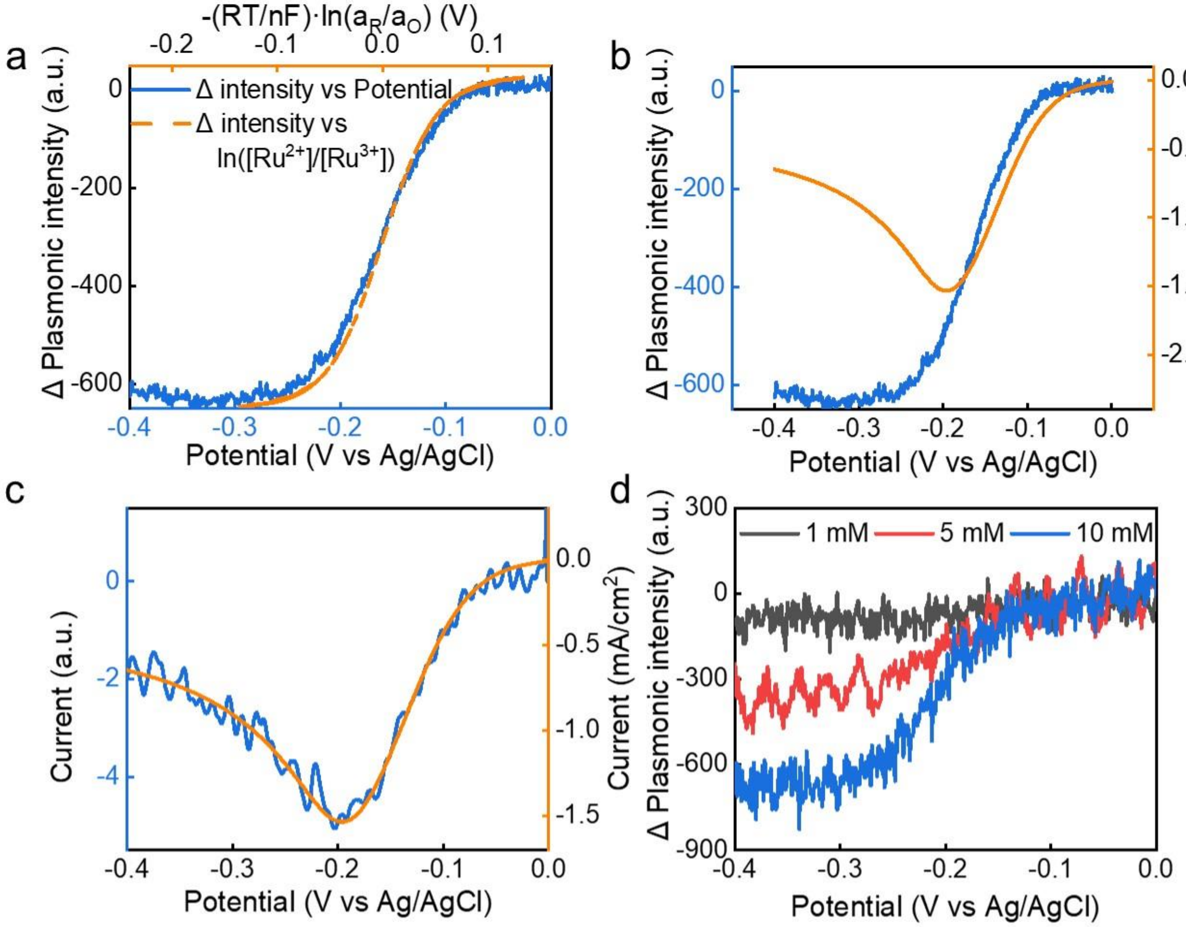

Supplementary Fig. 9 Correlation of the plasmonic intensity change (Δ*I*) and concentration change of the redox species. (a) The plasmonic intensity change curve vs. applied potential of the bare gold electrode (blue) and the simulated plasmonic intensity change curve vs. the concentration change of the redox species (orange). (b) The plasmonic intensity change curve vs. potential (blue) and the semi-integral of the conventional CV measured with an electrochemical workstation

(orange). (c) The current-potential curve calculated from plasmonic intensity (blue) and the conventional CV measured with an electrochemical workstation (orange). (d) Plasmonic intensity change curve vs. potential of monolayer MoS_2 on a 1-octadecanethiol-modified Au electrode with different concentrations of $[Ru(NH_3)_6]Cl_3$.

10. Determining the diffusion coefficient of $[Ru(NH_3)_6]^{2+}$ ions

By applying a constant potential of -0.3 V, we recorded the diffusion process of $[Ru(NH_3)_6]^{2+}$ ions, as shown in Supplementary Fig. 10. According to the three-dimensional semi-infinite diffusion model, the concentration of $[Ru(NH_3)_6]^{2+}$ ions as a function of diffusion distance (x) and time (t) is expected to follow the equation [10, 18]:

$$c(x,t) = \frac{M}{\sqrt{4\pi Dt}}\exp(-\frac{x^2}{4Dt})$$

where c is the concentration of the diffusing ions and M is the total mass of the diffusing ions. D is the diffusion coefficient. In the potentiostatic experiment, the diffusion of $[Ru(NH_3)_6]^{2+}$ ions reached the concentration peak when $x = 0$, $c_{max} = \frac{M}{\sqrt{4\pi Dt}}$, in less than 0.02 s. Therefore, the diffusion formula can be rewritten as:

$$\frac{c}{c_{max}} = \exp(-\frac{x^2}{4Dt})$$

Additionally, the concentration of $[Ru(NH_3)_6]^{2+}$ ions is proportional to its plasmonic intensity:

$$\Delta c = \beta \Delta I$$

where β is a constant. Bringing it into the diffusion formula, we obtain

$$\frac{\Delta I}{\Delta I_{max}} = \exp(-\frac{x^2}{4Dt})$$

The absolute distance x and the time interval between ΔI and ΔI_{max} can be measured by image pixels, and the diffusion coefficient of $[Ru(NH_3)_6]^{2+}$ ions was thus determined to be 4.8×10^{-10} m^2 s^{-1}, which was in accordance with the values in the literature[19].

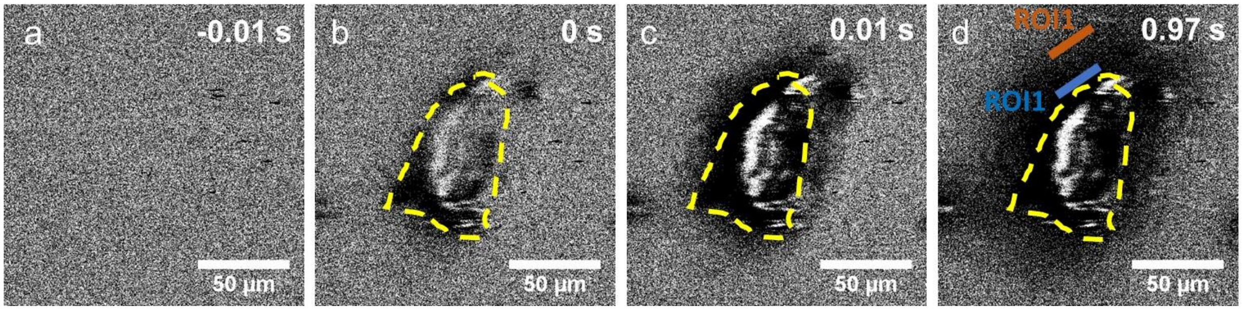

Supplementary Fig. 10 Diffusion of the $[Ru(NH_3)_6]^{2+}$ ions. (a) Difference plasmonic image at the open circuit voltage. (b-d) Snapshots of difference plasmonic images at a constant potential of -0.3 V, showing the diffusion of the $[Ru(NH_3)_6]^{2+}$ ions outward from monolayer MoS_2 to the surrounding solution. The yellow dashed line outlines the edge of monolayer MoS_2.

11. Conversion of the plasmonic intensity of single MoS_2 nanosheets to electrochemical current

The electrochemical current of monolayer MoS_2 was determined with the formula described previously[16, 20]. The quantitative relationship between the plasmonic signal and the electrochemical current of monolayer MoS_2 is given by

$$\Delta\theta(t) = A\int_0^t i\left(t'\right)\left(t-t'\right)^{-1/2} dt'$$

where $A = B\left(\alpha_R D_R{}^{-1/2} - \alpha_O D_O{}^{-1/2}\right)\left(nF\pi^{1/2}\right)^{-1}$ represents the concentration evolution of the reaction species from the electrochemical process inducing the plasmonic signal change[16]. Here, B is a constant of the experimental setup, α_R and α_O are the refractive index per unit concentration change of the reduced oxidized species that have been calibrated in Supplementary Note 9, D_R and D_O are the diffusion coefficients of the reduced and the oxides species, n is the number of electrons involved in the catalytic reaction, and F is the Faraday constant. In the reaction of the $[Ru(NH_3)_6]^{3+/2+}$ redox couple, A was calibrated to be 1.2388 RI/C with the experiments in Supplementary Note 9. With the Laplace transform performed on the equation above, the electrochemical current density $i(t)$ can be calculated from the plasmonic intensity, which is: $i(t) = nF\pi^{\frac{1}{2}}\left(B\left(\alpha_R D_R{}^{-1/2} - \alpha_O D_O{}^{-1/2}\right)\right)^{-1} L^{-1}(s^{\frac{1}{2}}\Delta\tilde{\theta}_{SPR}(s))$.

According to the calculation process described above, the calculated $\mathrm{i}(t)$ is the current derived from the layered MoS_2. Considering that the thiols could block the signals of the gold substrate, the measured $i(t)$ does not contain the double layer charging current. Therefore, the measured $i(t)$ merely reflects faradaic processes related to redox reactions.

12. Characterizations of MoS_2 for layer-dependent catalytic experiments

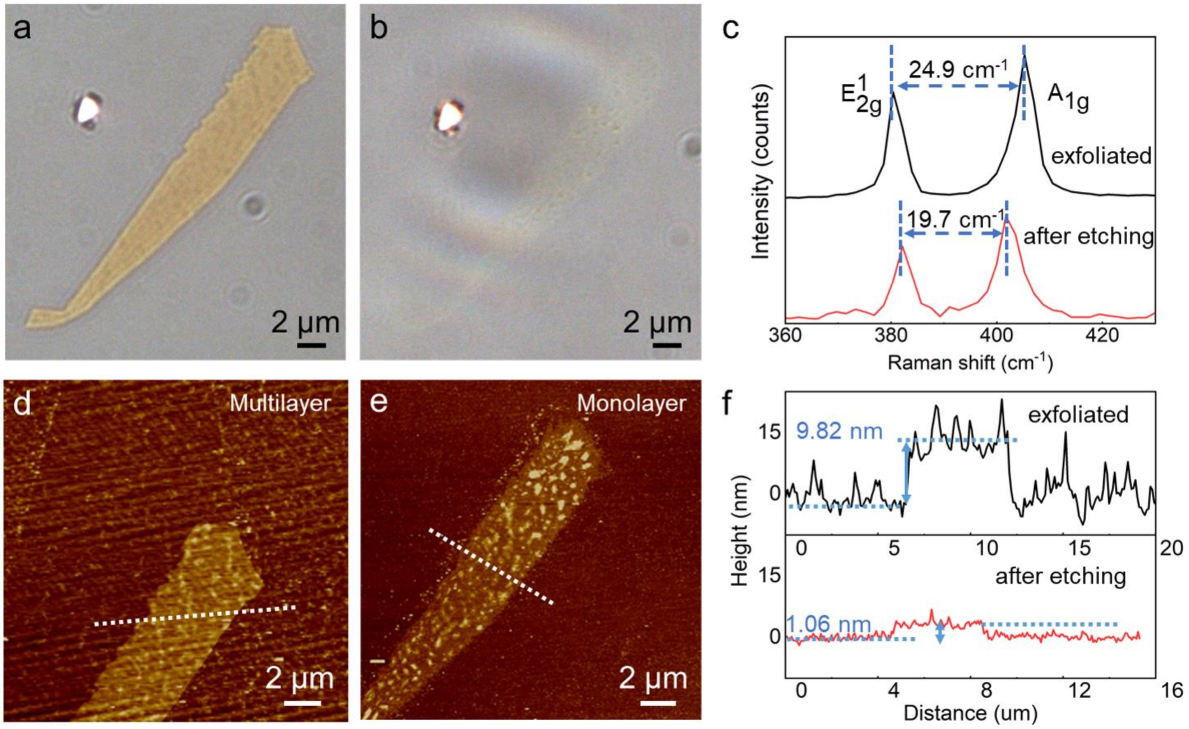

Supplementary Fig. 11 Characterization of the MoS_2 nanosheet used in the layer-dependent catalytic experiments. (a) Optical image of a pristine exfoliated MoS_2 nanosheet. (b) Optical image of monolayer MoS_2 after etching. (c) Raman spectra of the MoS_2 nanosheet after etching and of pristine exfoliated nanosheet for comparison. The MoS_2 nanosheet after etching shows a smaller peak difference between the E^1_{2g} and A_{1g} peaks (19.7 cm^{-1}) than those of the pristine MoS_2 nanosheet (24.9 cm^{-1}), suggesting the monolayer structure of MoS_2. (d-f) AFM images and corresponding height profiles of MoS_2 nanosheets before and after etching, indicating decreased thicknesses from 9.82 nm to 1.06 nm.

13. Catalytic kinetic analysis of MoS_2 with different thicknesses

To understand the relative rate of electron transfer at single MoS_2 nanosheets with different thicknesses, we extracted the half-wave potentials ($E_{1/2}$) of MoS_2 catalysing redox couples for kinetic comparison.

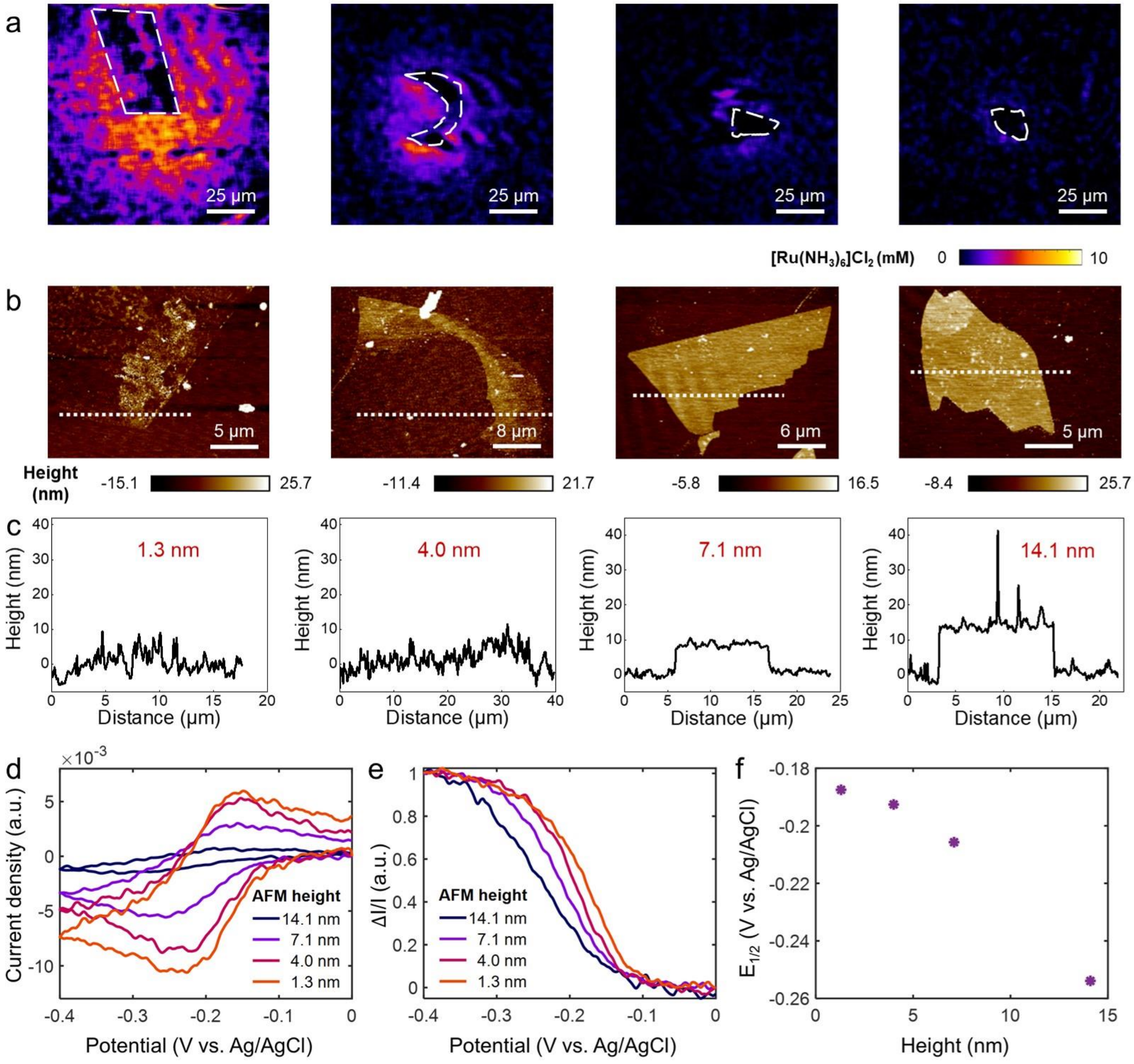

Supplementary Fig. 12 Mapping the electrocatalytic activity of layered MoS_2 with different thickness. (a) Plasmonic images of multilayer MoS_2 with different thicknesses at a potential of -0.4 V. (b-c) AFM images and corresponding height profiles of multilayer MoS_2 with different thicknesses. (d) Optical cyclic voltammograms of individual MoS_2 nanosheets. The scan rate was 100 mV s^{-1}. (e) Normalized plasmonic intensity responses of individual MoS_2 nanosheets with various thicknesses. (f) The voltammetric half-wave potential ($E_{1/2}$) shifts towards more positive

potentials as the thickness of MoS_2 decreases, further confirming that the rate of the $[Ru(NH_3)_6]^{3+/2+}$ redox process increases with decreasing numbers of layers.

14. Mapping the surface charge density of single MoS_2 nanosheets with various thicknesses

To unravel the layer-dependent electrocatalytic activity of the MoS_2 nanosheets, we mapped the surface charge density of single MoS_2 nanosheets with various thicknesses.

The calibration factor of the surface charge density and the plasmonic intensity is depended on the interfacial capacitances which is the layer-dependence C_Q, mainly. We firstly calculated their electron density-of-states (DOS) based on density functional theory (DFT) for further C_Q calculation. All DOS calculations of MoS_2 on Au (111) were carried out with DFT calculations using the CASTEP code, which has been widely applied to metal surfaces. All-electron calculations were employed using the generalized gradient approximation (GGA) and the Perdew, Bueke, and Ernzerhof (PBE) functional. In all calculations, the plane-wave cut-off energy was set to Fine mode, and variable occupancy calculations were conducted using the density mixing method. An electron smearing of 0.1 eV was used according to the Gaussian scheme. During the geometry optimization, the minimum energies for all structures were obtained until the energy became less than 1×10^{-5} eV/atom. The k-point sampling of the Brillouin zone was performed using a $1 \times 1 \times 1$ k-point grid for electronic structure computations. Mulliken charge analysis was performed to compute the charge transfer. The DOS of different-layer MoS_2 is shown in the Supplementary Fig. 13a. Then, we calculated the C_Q for different layers with the following equation:

$$C_Q = e^2 \int_{-\infty}^{+\infty} DOS(E)[(4kT)^{-1}\, sech^2(\frac{E-e\varphi}{2kT})]dE,$$

where the φ is the applied potential. Supplementary Fig. 13b shows the determined C_Q of MoS_2 with different number of layers.

Considering that the PBE functional has some limitations for the non-metallic systems, we also performed the DFT calculations using the HSE06 functional with the same conventional cell structure used above. Previous studies have proved that the results from PBE and HSE06 functionals can be mutually verified by calculating a variety of semiconductor materials[21, 22, 23, 24]. The HSE06 hybrid functional can perform better in band gap calculations for non-metal system. As shown in Supplementary Fig. 13a and c, the obtained DOSs from the two calculation methods show the same layer dependence whereas the HSE06 calculated bandgaps are slightly larger than the PBE calculated bandgaps. In our work, the band gap differences have little influence on the calculation of quantum capacitance. Hence, the quantum capacitances determined from both the HSE06 and PBE calculated DOSs are relatively close (Supplementary Fig. 13b and d).

The calibration factor for the conversion of plasmonic intensity to surface charge density varies with the number of layers. In manuscript Fig. 4g, we used the as-obtained layer-dependent C_Q values to calculate the calibration factor. The cited reference also used the DOS of MoS_2 to calculate C_Q, and our results were similar to that in the reference[9].

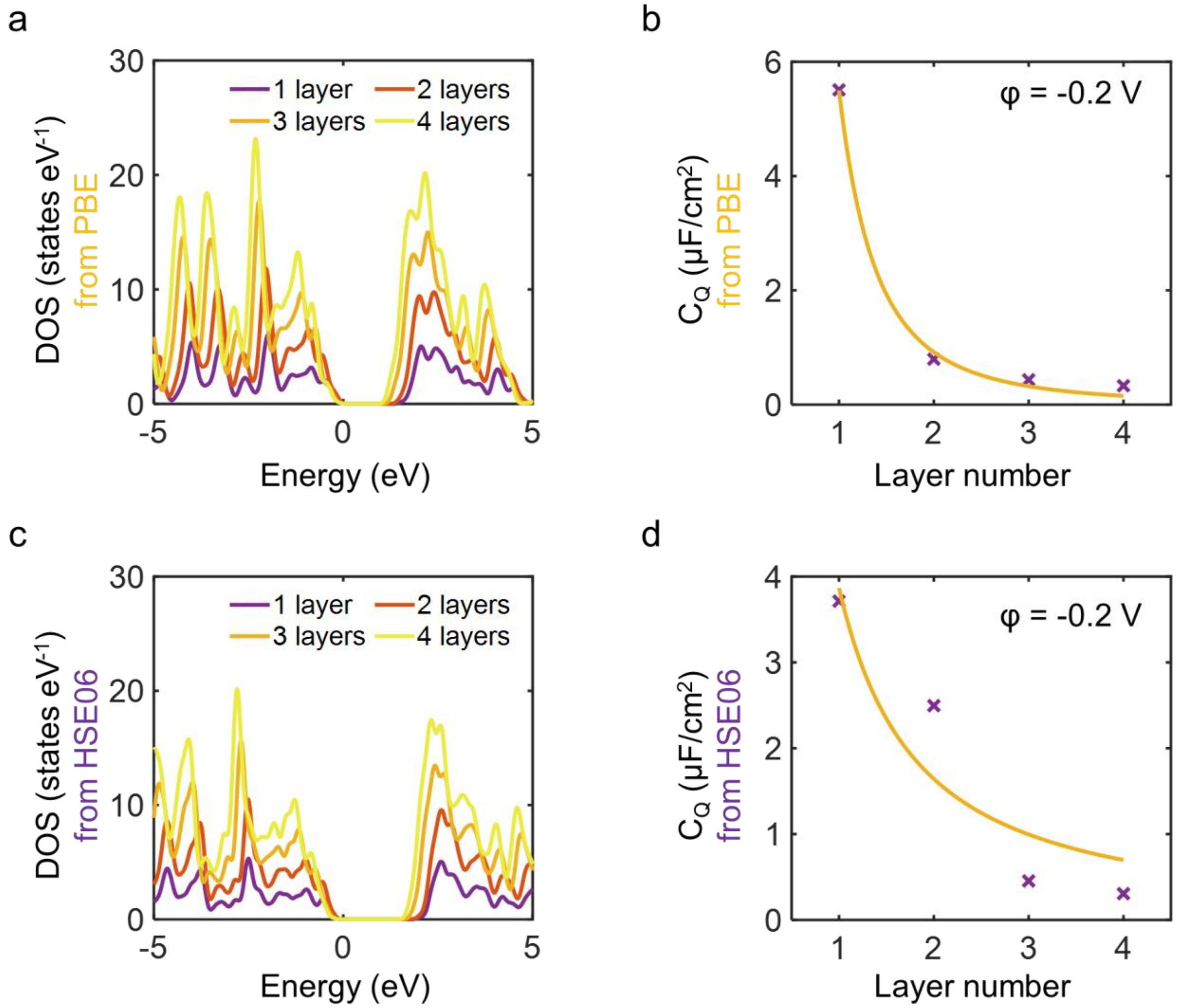

Supplementary Fig. 13 Theoretical calculations of layered MoS_2. (a-b) The calculated DOS and the calculated quantum capacitance of MoS_2 with different layers using the PBE functional. (c-d) The calculated DOS and the calculated quantum capacitance of MoS_2 with different layers using the HSE06 functional. The quantum capacitances were calculated at the applied potential of -0.2 V.

With the calculated calibration factor of the surface charge density and the plasmonic intensity, Supplementary Fig. 14 shows the surface charge density images and their corresponding

AFM images. We calculated the average surface charge density and plotted the curve of their thickness, as shown in manuscript Figure 4g. Having a stable double electrical capacitance in 0.1 M NaF solution, the background, bare gold substrate, displayed a surface charge density of ~4 $\mu C\ cm^{-2}$ in all the mapping results[25].

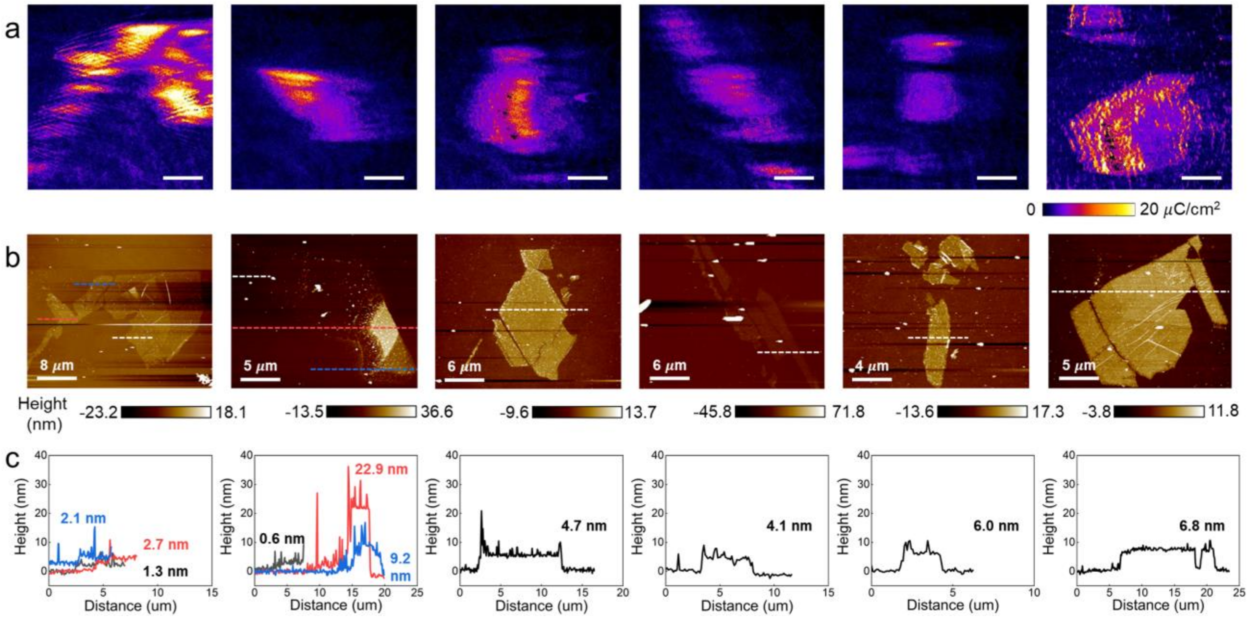

Supplementary Fig. 14 Mapping the surface charge density of layered MoS_2 with different thickness. (a) The surface charge density images, (b) their corresponding AFM images and (c) height profiles of the MoS_2 nanosheets with various thicknesses. Scale bars in the surface charge density images are 10 μm.

15. Au-MoS_2 Schottky contacts

In electrocatalytic process, the charge injection from the current collector to the catalyst and charge transport across the catalyst to the active sites determine the overall charge transport efficiency and thereby affect electrocatalytic activities. For semiconducting catalyst like MoS_2, a Schottky barrier exists at the metal–semiconductor interface to prevent efficient charge injection[26]. The Schottky barrier height is dictated by the energy difference between the work function of the metal and the conduction band edge of MoS_2. As shown in Supplementary Fig. 13, we depicted a band diagram at the Au–MoS_2 interface. As the layer number increases, upshift of conduction band edge in thicker MoS_2 decreases Schottky barrier height for electron injection. Therefore, the thicker MoS_2 has lower Schottky barrier height than the thinner one, leading to better electron transport ability in thicker MoS_2 at the Au–MoS_2 interface. However, we observed a contrary trend that the surface charge density of MoS_2 decreased quickly as its thickness increased in our work. That means, the electron transport across the interlayer of MoS_2 dominates the overall charge transport efficiency, rather than the charge transfer between MoS_2 and Au contact. Furthermore, the plasmonic imaging technique is sensitive to surface charge density, and the refractive index of MoS_2 decreased with the increase of the electron density. Consequently, the recorded contrasts in manuscript Figure 2 are originated from the voltage-controlled refractive index changes of MoS_2 which is highly related to the local charge density rather than the charge transfer between MoS_2 and Au contact.

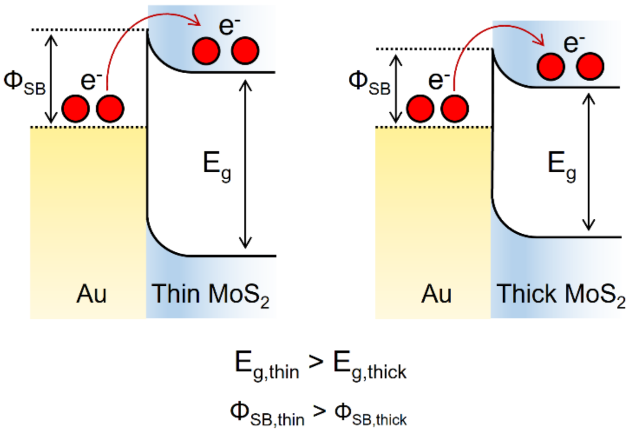

Supplementary Fig. 15 Au-MoS_2 Schottky contacts. Schematic energy band structures of the interfaces between contacted Au and MoS_2: thin MoS_2 (left) and thick MoS_2 (right). Φ_{SB} and E_g indicate Schottky barrier height and energy band gap of MoS_2, respectively.

16. Imaging the hydrogen evolution reaction (HER) at single MoS_2 nanosheets.

We measured the electrocatalytic hydrogen evolution reaction (HER) on single MoS_2 nanosheets by sweeping the electrode potential between 0 and -0.8 V (vs. Ag/AgCl) in 0.25 M H_2SO_4, and recorded the plasmonic images over time. The generated hydrogen molecule leads to the decrease in the local refractive index around MoS_2, which is reflected in the change of plasmonic image contrast. Supplementary Fig. 16a-b displays snapshots of differential plasmonic images at different potentials, where the image contrast decreases as the potential decreases, and achieves a maximum at the lowest potential. We selected a ROI around the MoS_2 and obtained the plasmonic image intensity curve in Supplementary Fig. 16c. Supplementary Fig. 16d presents the electrochemical current converted from the plasmonic signal change curve. The remarkable hydrogen evolution for MoS_2 takes place around -0.4 V, which is consistent with previous literature.

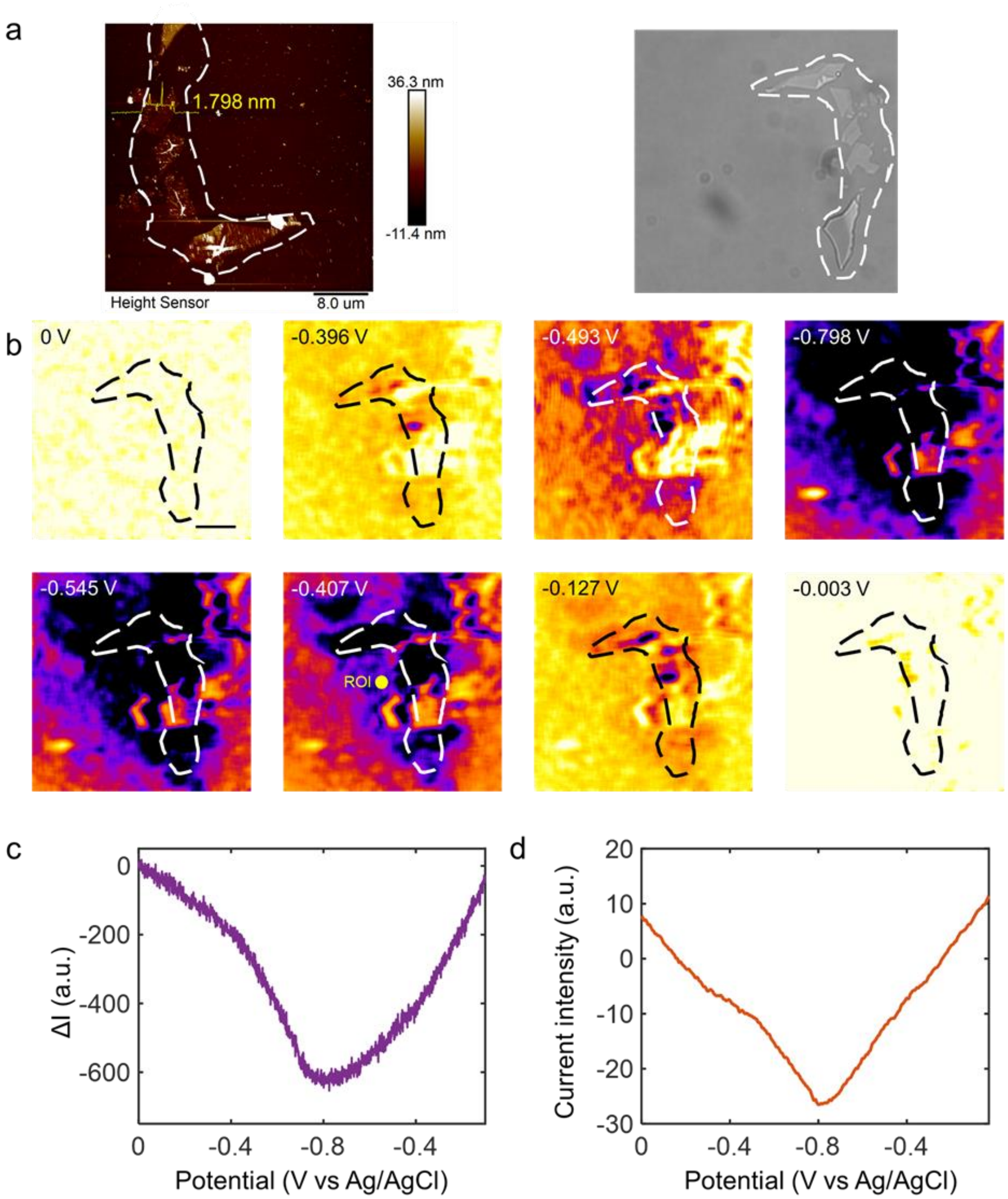

Supplementary Fig. 16 Imaging the hydrogen evolution reaction (HER) process at single MoS_2 nanosheets. (a) AFM and wide-field optical images of the MoS_2 nanosheets. (b) Snapshots of differential plasmonic images during the potential sweeping in 0.25 M H_2SO_4. Potential sweeping rate: 100 mV s^{-1}. Scale bar: 5 μm. (c) The plasmonic intensity change ($\Delta I_{HER}/I$) vs. potential and (d) corresponding cyclic voltammogram converted from the plasmonic signal change curve.

17. Layer-dependent electrocatalytic activity of other two-dimensional materials

Our imaging method is facile and can be applied to probe the electrocatalytic activity of other two-dimensional (2D) materials. We also imaged the electrocatalytic activity of three other kinds of 2D materials with the quantitative method in Supplementary Note 11, including graphene, WS_2 and $MoSe_2$, which exhibit similar layer-dependent electrocatalytic behaviours. Supplementary Fig. 17a-c display the cyclic voltammograms of graphene, WS_2 and $MoSe_2$ with various thicknesses determined by our electrochemical imaging technique. All of the materials exhibited faster electron transfer kinetics with decreased layer numbers due to the decreased interlayer electron transfer ability as the number of layers increases. Similar to MoS_2, the half-wave potentials of graphene and WS_2 shifted towards more positive potentials with decreasing the number of layers (Supplementary Fig. 17d). Meanwhile, CV curves of $MoSe_2$ did not show significant redox peaks in the potential range. The electron transfer kinetic of $MoSe_2$ is much slower than that of MoS_2 and WS_2 under the same conditions. In general, electron transfer between interlayers in these vdW materials occurs via electron tunneling, and the interlayer conductivity can decrease significantly as its thickness increases[27, 28, 29]. These results suggest that interlayer electron transport plays a considerable role in the electrocatalysis of 2D materials.

Given the layer-dependent electrochemical activity of these 2D nanomaterials, it is noteworthy that only the materials with the same layer number can compare their electrocatalytic and electrochemical properties. We further compared the electrochemical response of different 2D nanomaterials with similar layer numbers (~ 8 layers). As shown in Supplementary Fig. 17e, the maximum reduction current density of graphene is larger than the other three nanomaterials, indicating a more facile electron transfer kinetic of graphene under the same number of layers. This is because graphene is a semi-metal with a high electrical conductivity and the other three TMDs are semiconducting catalysts[30, 31]. However, the high electrical conductivity of a catalyst does not promise a high catalytic performance since graphene is a catalytic inert material. This is because the adsorption/desorption kinetics of the reactants on the catalyst active sites is another important factor. They both contribute to the catalytic performance of a catalyst.

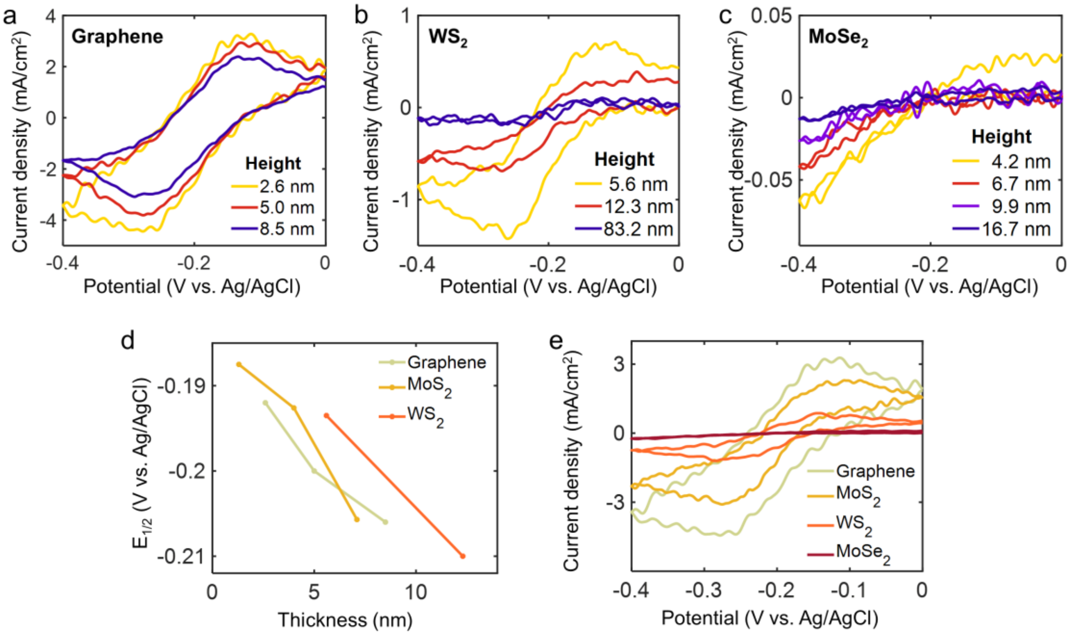

Supplementary Fig. 17 The application of the plasmonic imaging method to probe the electrocatalytic activity of other 2D materials. CV curves of (a) graphene, (b) WS_2 and (c) $MoSe_2$ with various thicknesses determined by our electrochemical imaging technique. (d) The half-wave potentials change with the thickness of Graphene, MoS_2, and WS_2. (e) The CV curves of different materials with about 8 layers.

References

1. Santos EJG, Kaxiras E. Electrically Driven Tuning of the Dielectric Constant in MoS_2 Layers. *ACS Nano* **7**, 10741-10746 (2013).
2. Yu Y, Yu Y, Huang L, Peng H, Xiong L, Cao L. Giant Gating Tunability of Optical Refractive Index in Transition Metal Dichalcogenide Monolayers. *Nano Lett* **17**, 3613-3618 (2017).
3. Foley KJ, Shan X, Tao NJ. Surface Impedance Imaging Technique. *Analytical Chemistry* **80**, 5146-5151 (2008).
4. Homes CC, Ali MN, Cava RJ. Optical properties of the perfectly compensated semimetal WTe_2. *Physical Review B* **92**, 161109 (2015).
5. Mannebach EM, *et al.* Dynamic Optical Tuning of Interlayer Interactions in the Transition Metal Dichalcogenides. *Nano Letters* **17**, 7761-7766 (2017).
6. Born M, Wolf E. *Principles of optics: electromagnetic theory of propagation, interference and diffraction of light*. Elsevier (2013).
7. Kravets VG, Wu F, Auton GH, Yu T, Imaizumi S, Grigorenko AN. Measurements of electrically tunable refractive index of MoS_2 monolayer and its usage in optical modulators. *npj 2D Materials and Applications* **3**, 36 (2019).
8. Martino FA. Effect of Electrolyte Concentration on the Capacitance and Mobility of Graphene. In: *Physics*). Linfield University (2016).
9. Fang N, Nagashio K. Band tail interface states and quantum capacitance in a monolayer molybdenum disulfide field-effect-transistor. *Journal of Physics D: Applied Physics* **51**, 065110 (2018).
10. Bard AJ, Faulkner LR. *Electrochemical methods: Fundamentals and applications*. John Wiley & Sons, Inc. (2001).
11. Dieter Britz JS. *Digital Simulation in Electrochemistry*, 4 edn. Springer Cham (2016).
12. Lu J, Wang W, Wang S, Shan X, Li J, Tao N. Plasmonic-Based Electrochemical Impedance Spectroscopy: Application to Molecular Binding. *Analytical Chemistry* **84**, 327-333 (2012).
13. He Y, *et al.* Self-gating in semiconductor electrocatalysis. *Nat Mater* **18**, 1098-1104 (2019).
14. Xu Q, Yang GM, Zheng WT. DFT calculation for stability and quantum capacitance of MoS_2 monolayer-based electrode materials. *Materials Today Communications* **22**, 100772 (2020).
15. Choma MA, Sarunic MV, Yang C, Izatt JA. Sensitivity advantage of swept source and Fourier domain optical coherence tomography. *Opt Express* **11**, 2183-2189 (2003).
16. Shan X, Patel U, Wang S, Iglesias R, Tao N. Imaging local electrochemical current via surface plasmon resonance. *Science* **327**, 1363-1366 (2010).
17. Reynolds MF, *et al.* MoS_2 pixel arrays for real-time photoluminescence imaging of redox molecules. *Sci Adv* **5**, eaat9476 (2019).
18. Coifman RR, *et al.* Geometric diffusions as a tool for harmonic analysis and structure definition of data: Diffusion maps. *Proc Natl Acad Sci U S A* **102**, 7426-7431 (2005).
19. Wang Y, Limon-Petersen JG, Compton RG. Measurement of the diffusion coefficients of $[Ru(NH_3)_6]^{3+}$ and $[Ru(NH_3)_6]^{2+}$ in aqueous solution using microelectrode double potential step chronoamperometry. *Journal of Electroanalytical Chemistry* **652**, 13-17 (2011).
20. Wang S, Huang X, Shan X, Foley KJ, Tao N. Electrochemical Surface Plasmon Resonance: Basic Formalism and Experimental Validation. *Analytical Chemistry* **82**, 935-941 (2010).
21. Perdew JP, Burke K, Ernzerhof M. Generalized Gradient Approximation Made Simple. *Physical Review Letters* **77**, 3865-3868 (1996).
22. Ding Y, Wang Y, Ni J, Shi L, Shi S, Tang W. First principles study of structural, vibrational and electronic properties of graphene-like MX_2 (M=Mo, Nb, W, Ta; X=S, Se, Te) monolayers. *Physica B: Condensed Matter* **406**, 2254-2260 (2011).
23. Zhang G-X, Reilly AM, Tkatchenko A, Scheffler M. Performance of various density-functional approximations for cohesive properties of 64 bulk solids. *New Journal of Physics* **20**, 063020 (2018).

24. Singh A, Dey M, Singh AK. Origin of layer-dependent electrical conductivity of transition metal dichalcogenides. *Physical Review B* **105**, 165430 (2022).
25. Shan X, *et al.* Mapping Local Quantum Capacitance and Charged Impurities in Graphene via Plasmonic Impedance Imaging. *Adv Mater* **27**, 6213-6219 (2015).
26. Schulman DS, Arnold AJ, Das S. Contact engineering for 2D materials and devices. *Chemical Society Reviews* **47**, 3037-3058 (2018).
27. Chen R-S, Tang C-C, Shen W-C, Huang Y-S. Thickness-dependent electrical conductivities and ohmic contacts in transition metal dichalcogenides multilayers. *Nanotechnology* **25**, 415706 (2014).
28. Fang X-Y, Yu X-X, Zheng H-M, Jin H-B, Wang L, Cao M-S. Temperature- and thickness-dependent electrical conductivity of few-layer graphene and graphene nanosheets. *Physics Letters A* **379**, 2245-2251 (2015).
29. Kim H-C, *et al.* Engineering Optical and Electronic Properties of WS2 by Varying the Number of Layers. *ACS Nano* **9**, 6854-6860 (2015).
30. Geim AK, Novoselov KS. The rise of graphene. *Nature Materials* **6**, 183-191 (2007).
31. Kang W, Wang Y, Xu J. Recent progress in layered metal dichalcogenide nanostructures as electrodes for high-performance sodium-ion batteries. *Journal of Materials Chemistry A* **5**, 7667-7690 (2017).
