# Supplementary material for: Plasmonic imaging of the layer-dependent electrocatalytic activity of two-dimensional catalysts
Source: Nat Commun. 2022 Dec 22;13:7869. doi: 10.1038/s41467-022-35633-3 (PMC9780338; doi:10.1038/s41467-022-35633-3)
Supplement: Supplementary file 2 — Description of Additional Supplementary Files [file 41467_2022_35633_MOESM2_ESM.pdf]

Description of Additional Supplementary Files

File Name: Supplementary Movie 1
Description: A video showing the electrochemical reaction of [Ru(NH3)6]Cl3 on monolayer MoS2 during continuous cycling of the potential between -0.4 V and 0 V at a rate of 100 mV s-1.

File Name: Supplementary Movie 2
Description: A video showing the layer-dependent electrocatalytic activity of single MoS2 nanosheets (up: multilayer MoS2; down: monolayer MoS2). A higher image contrast was observed for monolayer MoS2, which had a larger diffusion area of [Ru(NH3)6]2+ than multilayer MoS2.
